# Orofacial and cervical myofunctional intervention programs for adults undergoing orthognathic surgery: a scoping review

**DOI:** 10.1590/2317-1782/e20240358en

**Published:** 2025-11-28

**Authors:** Allya Francisca Marques Borges, Ramon Cipriano Pacheco de Araújo, Samara Fernandes da Silva Souza, Aline Xavier Ferraz, Cristiano Miranda de Araújo, Hipólito Virgílio Magalhães, Renata Veiga Andersen Cavalcanti, Karinna Veríssimo Meira Taveira

**Affiliations:** 1 Programa de Pós-graduação em Fonoaudiologia, Universidade Federal do Rio Grande do Norte – UFRN - Natal (RN), Brasil.; 2 Grupo de Pesquisa Estudos em Motricidade Orofacial e Disfagia Orofaríngea, Universidade Federal do Rio Grande do Norte – UFRN - Natal (RN), Brasil.; 3 Programa de Pós-graduação em Distúrbios da Comunicação, Núcleo de Estudos Avançados em Revisão Sistemática e Meta-análise – NARSM, Universidade Tuiuti do Paraná – UTP - Curitiba (PR), Brasil.; 4 Departamento de Fonoaudiologia, Universidade Federal do Rio Grande do Norte – UFRN - Natal (RN), Brasil.; 5 Programa Associado de Pós-graduação em Fonoaudiologia, Núcleo de Estudos Avançados em Revisão Sistemática e Meta-análise – NARSM, Departamento de Morfologia, Centro de Biociências, Universidade Federal do Rio Grande do Norte – UFRN - Natal (RN), Brasil.

**Keywords:** Myofunctional Therapy, Dentofacial Deformities, Orthognathic Surgery, Stomatognathic System, Rehabilitation

## Abstract

**Purpose:**

To map orofacial and cervical myofunctional intervention programs developed for adults undergoing orthognathic surgery.

**Research strategies:**

Search of Cochrane, EMBASE, LILACS, LIVIVO, PubMed/Medline, Scopus, and Web of Science databases, as well as gray literature.

**Selection criteria:**

The review included studies addressing therapeutic programs and myofunctional exercises and their influence on the stomatognathic system of adults undergoing orthognathic surgery, without time or language limitations. Secondary studies, populations under 18 years old, and comorbidities associated with dentofacial deformities were excluded.

**Data analysis:**

Two reviewers extracted data comprising different topics, especially the assessment process, orofacial and cervical myofunctional intervention, and identification of intervention outcomes.

**Results:**

Five studies were considered eligible. They were published between 2006 and 2018, with participants aged 19 to 31, predominantly females, and used postoperative guidelines consisting of isometric, isotonic, postural, and functional exercises, as well as tactile-kinesthetic and thermal stimulation, with a positive emphasis on functional improvement of the stomatognathic system.

**Conclusion:**

These studies addressed the application of orofacial myofunctional therapy after orthognathic surgery. The interventions, not yet validated, involved specific protocols of postural and functional exercises and tactile-kinesthetic and thermal stimulation. The results demonstrated a significant improvement in the performance of orofacial functions, enhanced subjective perception of oral health, and improvements in muscle tone and mobility of orofacial structures. Orofacial myofunctional therapy is an important approach in postoperative rehabilitation of the stomatognathic system.

## INTRODUCTION

Dentoskeletal or dentofacial deformity (DFD) is a condition that manifests as disharmony between the teeth and jaws, combining dental and skeletal malocclusion^(1)^. The arrangement of the bone and dental bases influences the adjacent soft tissues, which can have a significant impact on the individual's quality of life and orofacial functions^(1,2)^.

Facial pattern restoration through the correction of mandibular, maxillary, and/or mental region disproportions and facial asymmetries, after the end of craniofacial growth and development, requires orthognathic surgery associated with orthodontic treatment^(2-4)^.

The combination of orthognathic surgery and orthodontic treatment enables effective correction of facial asymmetries, establishing a harmonious balance between the teeth, supporting bones, and adjacent facial structures^(4)^. However, altered functional patterns persist in some cases, which can compromise the maintenance of orofacial myofunctional balance^(5)^. The latter can be achieved through orthosurgical treatment to ensure stable treatment results, requiring an interdisciplinary approach and structured orofacial myofunctional therapy (OMT)^(5-7)^.

OMT encompasses a set of techniques based on specific exercises targeting the orofacial and cervical muscles and orofacial functions to develop proprioception, sensitivity, mobility, coordination, and strength of the functions and structures of the stomatognathic system (SS)^(8)^. The approach primarily aims to optimize the SS vital functions, including chewing, swallowing, sucking, speaking, and breathing^(5,7,9)^.

Orthognathic surgery plays an important role in restoring the balance of orofacial structures in individuals with DFD, which involves reconfiguring the proprioceptive scheme so that soft tissue structures perform their functions satisfactorily^(7,10,11)^. Hence, it is essential to investigate the impact of OMT on the SS structural and functional aspects before and after DFD surgical correction. Detailed analysis will provide valuable insights into the adaptation of orofacial muscles and functions after surgical intervention, contributing significantly to the development of more effective and personalized therapeutic approaches for individuals with DFD undergoing orthognathic surgery, as well as reducing relapses due to persistent inadequate functional patterns.

From the perspective of SS functional and structural balance in individuals with DFD, this scoping review was conducted to provide a more comprehensive insight into this knowledge, map the existing literature, examine how research is conducted in this field, analyze knowledge gaps, and suggest future research^(12)^. Thus, this review aimed to map the synthesis of evidence on orofacial and cervical myofunctional intervention programs developed for adults undergoing orthognathic surgery and their influence on the SS.

## METHODS

This scoping review was conducted in strict accordance with the guidelines outlined in the Joanna Briggs Institute (JBI) manual^(13)^. The protocol and review were developed using the Preferred Reporting Items for Systematic Reviews and Meta-Analyses – Extension for Scoping Reviews (PRISMA-ScR) checklist^(14)^, thus ensuring a transparent process. The protocol for this study was registered with the Open Science Framework registry platform^(15)^.

### Eligibility criteria

The acronym “PCC” (Participants, Concept, Context) was used to consider the eligibility of studies to be included in or excluded from this review, as follows: Population (P): adults with DFD; Concept (C): OMT; Context (C): orthognathic surgery.

### Inclusion criteria

This review included studies that fully described the orofacial and cervical myofunctional intervention programs developed for adults undergoing orthognathic surgery; that analyzed the proposed actions and exercises and their impact on chewing, swallowing, sucking, speech, breathing, and facial muscles of adults with DFD undergoing orthognathic surgery; that considered the form of pre- and post-intervention assessment of orofacial functions; that reported the surgical period (pre-surgical, immediate post-surgical, or late post-surgical) when the exercises or actions were performed; and studies with males and females aged 18 years or older, without time and language delimitation.

### Exclusion criteria

The following were excluded from this review: 1. Studies in which health professionals other than speech-language-hearing pathologists performed orofacial and cervical myofunctional intervention. 2. Studies with adults with a history of psychiatric, neurological, neuromuscular, or neurodegenerative and oncological disorders in the orofacial and/or cervical region. 3. Studies that included adults with cleft lip and palate, oropharyngeal dysphagia, obstructive sleep apnea syndrome, temporomandibular disorder, and/or facial trauma. 4. Secondary studies such as abstracts, systematic reviews, narrative reviews, integrative reviews, meta-analyses, letters to the editor, guidelines, and clinical practice guidelines. 5. Studies with repeated samples.

### Sources of information and search strategy

Words were combined and truncated according to each electronic database, including Cochrane, EMBASE, Latin American and Caribbean Health Sciences Literature (LILACS), LIVIVO, PubMed/Medline, Scopus, and Web of Science. Gray literature was also used as a source of information through the Brazilian Digital Library of Theses and Dissertations (BDTD), Google Scholar, and ProQuest Theses and Dissertations. Furthermore, the references of the included studies were manually searched, and an expert was consulted to verify any possible missing articles. The literature survey was conducted on October 25, 2023. An experienced reviewer (KVMT) developed search strategies, which were refined through team discussion. The final search strategy for each database and gray literature is demonstrated in Appendix A. The search results were exported to Endnote® software (EndNote® X7 Thomson Reuters, Philadelphia, PA).

### Study selection

Two independent reviewers (AFMB and SFSS) read the titles, abstracts, and full texts, using Rayyan®️software^(16)^. In case of doubts or conflicts, a third independent reviewer (KVMT) was consulted.

They were calibrated before title and abstract analysis by selecting 100 initial references retrieved from the literature^(17)^. The calibration aimed for a value > 0.7 in the kappa concordance coefficient before beginning data selection and extraction^(17)^.

### Data extraction

Two reviewers (AFMB and SFSS) extracted data, with additional consultation with two other reviewers (KVMT and RVAC) to clarify doubts related to the extracted variables, study designs, and the classification of OMT programs and orofacial exercises. The categorization of the studies, presented in Table 1, included their general information, such as title, author, year, country, study design, study objective, methodological characteristics (sample and age), evaluation (instruments analyzed and temporal parameters), DFD classification, surgical period when myofunctional exercises were performed, and intervention (classification according to the type of program or exercises and their frequency). Table 1 also presents a summary of the outcomes, highlighting the functional and muscular effects resulting from the interventions. When the data were incomplete, three attempts were made to contact the authors (first, last, and corresponding author) to obtain this information; if there was no response, the article was excluded.

**Table 1:** Characterization of studies that analyzed the impact of orofacial myofunctional therapy in volunteers undergoing orthognathic surgery (n = 5)

**Author, Year, Country, Study Design**	**Objective**	**Methodological characteristics**		**Assessment**		**Classification of dentofacial deformity**	**Time since surgery**	**Intervention**			**Main outcomes**
		**Sample**	**Age**	**Instruments used**	**Temporal parameters**			**Programs/Exercises**	**Frequency**		
Migliorucci et al.^(5)^, Brazil, Randomized clinical trial	To verify the impact of myofunctional therapy on orofacial functions and the quality of life of patients undergoing orthognathic surgery.	OMT group: 12 (7F, 5M)	± 26.35	Orofacial Myofunctional Assessment Protocol (MBGR) and Oral Health Impact Profile-OHIP-14	Before the surgical procedure and 3 months after orthognathic surgery.	Class II: 5 Class III: 7	The intervention began 40 days after surgery.	Tactile-kinesthetic and thermal stimulation. Isometric, isotonic, and functional exercises	Once a week for 8 to 15 weeks		Significant improvement in orofacial functions, such as breathing (p=0.005), chewing (p=0.006), swallowing (p=0.003), and speech (p=0.012). Positive impact on quality of life, according to OHIP-14 (p=0.018)
		No-OMT group: 12 (7F, 5M)	± 26.35			Class II: 5 Class III: 7		No participation in the exercise program.			Significant improvement in breathing (p=0.044) and quality of life, according to the OHIP-14 (p=0.003). No improvement in chewing (p=0.757), swallowing (p=0.060), or speech (p=0.539) parameters
Prado et al.^(11)^, 2018, Brazil, Randomized clinical trial	To investigate the effects of orofacial myofunctional therapy on the masticatory function of individuals with dentofacial deformities undergoing orthognathic surgery.	TG (OMT): 13 (7F, 6M)	29.31 (± 8.87)	Orofacial Myofunctional Evaluation Protocol with Scores-extended (OMES-E) and electromyography	Before and after the surgical procedure (3 and 6 months)	Class II: 4 Class III: 9	The intervention began 30 days after surgery	Tactile-kinesthetic and thermal stimulation. Isometric, isotonic, postural, and functional exercises.	Once a week for 10 weeks.		Increase in the maximum OMES-E score from P0 (13.23 ± 3.06) to P1 (15.92 ± 3.84) and P2 (16.0 ± 3.51), and from P0 (4.3 ± 2.56) to P2 (6.92 ± 2.78). The number of masticatory cycles increased from P0 (11.34 ± 2.87) to P2 (13.79 ± 2.44). Lip tone increased from P0 (0 - 0%) to P1 (6 - 46.15%) and P2 (8 - 61.54%), and mobility from P0 (4 - 30.77%) to P1 and P2 (11 - 84.61%). Bite, head movements, food spillage, and tongue tone did not differ significantly. Abnormal head posture persisted in 2 to 8 individuals.
		UTG (OMT): 10 (7F, 3M)	31.20 (± 7.02)			Class II: 7 Class III: 3	The intervention began 6 months after surgery, after all initial assessments were completed.	The group did not participate in the exercise program throughout the evaluation period. The intervention occurred after the study was completed.			Bite did not differ significantly between the periods, but there were more individuals with abnormal lower lip tone and lip and tongue mobility.
Trawitzki et al.^(18)^, 2006, Brazil, Cohort study	To determine the effect of interdisciplinary treatment in a patient with Class III dentofacial deformities by analyzing the electromyographic activity of the temporal and masseter muscles during chewing and biting.	P1 group**: 15 (4M, 11F)	M: ± 21.2 years F: ± 24.9 years	Ultrasound	3 months before orthognathic surgery*	Class III	The intervention started 15 days after surgery and lasted 6 months*	Group P1 is identical to group P2. However, it is being used as a reference for pre-surgical evaluation in group P2.			There are no results to be analyzed.
		P2 group**: 15 (4M, 11F)	M: ± 21.2 years F: ± 24.9 years		6 to 8 months after orthognathic surgery*	Corrected Class III		Post-surgical guidance. Isometric, isotonic, postural, and functional exercises. Tactile and thermal sensory stimulation*	Once a week for 6 months.		Significant increase in masseter thickness in P2, both at rest and when biting, compared to P1. However, P2 values ​​remained lower than those of the control group, bilaterally.
		Control group: 15 (4M, 11F)	M: ± 20.8 years F: ± 24 years		Before and after orthognathic surgery, in the periods mentioned in the experimental groups.	Absence of dentofacial deformity	OMT not performed	No participation in the exercise program.			Higher values ​​with statistical significance, when compared to P1 and P2, regarding the thickness of the masseter muscle, bilaterally.
Trawitzki et al.^(19)^, 2011, Brazil, Cohort study	To analyze the effect of integrated orthodontic treatment, orthognathic surgery, and orofacial myofunctional therapy on the thickness of the masseter muscle in patients with class III dentofacial deformities, 3 years after orthognathic surgery.	P1 group**: 13 (3M, 10F)	M: ± 22 years F: ± 27 years	Ultrasound	2 to 74 days before orthognathic surgery.	Class III	The intervention started 15 days after surgery and lasted 6 months.	Group P1 is identical to group P3. However, it is being used as a reference for pre-surgical evaluation in group P3.			There are no results to be analyzed.
		P3 group**: 13 (3M, 10F)	M: ± 22 years F: ± 27 years		Data were obtained over 3 years to 3 years and 8 months postoperatively (3 years and 2 months).	Corrected Class III		Post-surgical guidance. Isometric, isotonic, postural, and functional exercises. Tactile and thermal sensory stimulation*	Once a week for the first 11 months after surgery.		Significant increase in the thickness of the right and left masseter (P < 0.01). In comparison with the control group, a significant difference was observed for the right masseter at rest (P < 0.01) and when biting (P < 0.05), and for the left masseter at rest (P < 0.05). There was no significant difference for the left masseter when biting (P = 0.05).
		Control group: 15 (4M, 11F)	M: ± 21 years F: ± 24 years		Before and after orthognathic surgery, in the periods mentioned in the experimental groups.	Absence of dentofacial deformity	OMT not performed.	No participation in the exercise program.			There are no results to report.
Trawitzki et al.^(20)^, 2010, Brazil, Cohort study	To investigate the effect of interdisciplinary treatment on the electromyographic activity of the masticatory muscles in patients with Class III dentofacial deformities, 3 years after surgical correction.	P1 group***:13 (3M, 10F)	M: ± 22 years F: ± 27 years	Electromyography	Before the surgical procedure.	Class III	The intervention started 15 days after surgery and lasted 6 months.	Group P1 is identical to group P3. However, it is being used as a reference for pre-surgical evaluation in group P3.			In the T and M muscles, there were significant differences between the P1 and P3 Groups, in chewing and biting, with higher values ​​in P3***
		P3 group***:13 (3M, 10F)	M: ± 22 years F: ± 27 years		Data were collected over 3 years to 3 years and 8 months postoperatively (3 years and 2 months).	Corrected Class III		Post-surgical guidance. Isometric, isotonic, postural, and functional exercises. Tactile and thermal sensory stimulation*		It lasted an average of 11 months.	In EMG activity, there was a significant disparity (P < 0.05) in the values ​​of the M muscle during chewing and biting, with higher values ​​in P3***
		Control group: 15 (11F, 4M)	M: ± 21 years F: ± 24 years		Before and after orthognathic surgery, in the periods mentioned in the groups.	Absence of dentofacial deformity.	OMT not performed.	No participation in the exercise program.			Statistically significant difference in EMG activity for M and T muscles in different situations

Caption: F = female; M = male; OMT = orofacial myofunctional therapy; TG = treated group; UTG = untreated group; P0 = before surgery; P1 = 3 months after surgery; P2 = 6 months after surgery; P1** = before surgery; P2** = 6 months after surgery; P1*** = before surgery; P3*** = postoperative (3 years to 3 years and 8 months); OHIP-14 = Oral Health Impact Profile; M = Masseter muscle; T = Temporalis muscle

* Information provided by the author of the included study^(18)^

### Data analysis

Data were analyzed to map orofacial and cervical myofunctional intervention programs developed for adults with DFD undergoing orthognathic surgery and verifying their influence on the SS.

Speech-language-hearing therapy in the postoperative period of orthognathic surgery is to reduce facial edema, promote orofacial sensitivity and facial expressions, increase the range of mandibular movements, and gradually reintroduce food consistencies and rehabilitate orofacial functions^(7)^. Hence, clinical practice uses OMT with isometric (static contraction), isotonic (dynamic contraction), and counter-resistance exercises.

Therapeutic planning consists of the repetition and systematic practice of these exercises, with a gradual approach in terms of frequency, volume (number of sets, repetitions, and sessions), and rest intervals. Therefore, the analysis of the extracted variables aimed to examine three main outcomes: type of exercise (isometric, isotonic, or counter-resistance), functional effects (orofacial functions, quality of life, and oral health), and muscular effects (tone and mobility).

Following data extraction, the results were analyzed in two stages: study categorization and analysis of outcomes. This constituted a qualitative and descriptive process, given the heterogeneous nature of the included studies.

**Figure 1:**
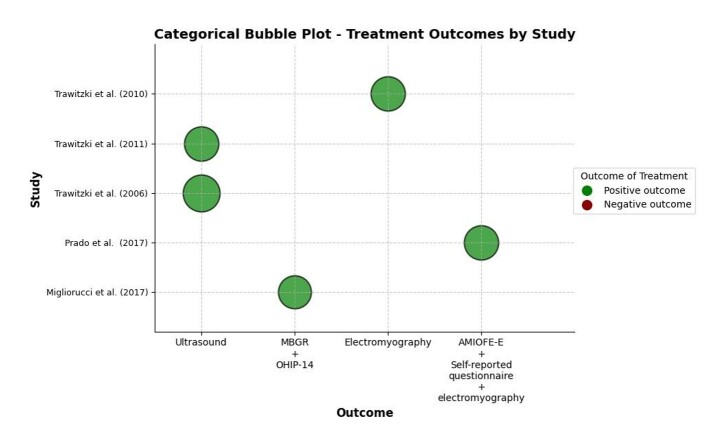
Bubble plot for categorical variables for the outcome of orofacial myofunctional therapy in adults undergoing orthognathic surgery

The analysis of the results presented in the evaluation, whether using instrumental methods, orofacial myofunctional assessment protocols, or self-assessment questionnaires, was presented graphically in a bubble plot suitable for visualizing categorical variables, as illustrated in Figure 1. It was constructed with Python and the Matplotlib and NumPy libraries for data manipulation and graphical visualization. The evaluation result was considered positive (with improvement in the SS structural and functional patterns during the post-orthognathic surgery period, represented by green bubbles) or negative (with worsening of the SS structural and functional patterns during the post-orthognathic surgery period, represented by red bubbles). Each study was represented by a bubble, positioned on the x- and y-axes to indicate, respectively, the study strategy and author. The color of the bubbles was used to differentiate the results, and their size indicates the sample of individuals who underwent OMT. Thus, the graph provides a clear, comparative, visual representation of the results of the studies included in this review.

Reasons for exclusion:

Reason 1. Studies in which health professionals other than speech-language-hearing pathologists performed orofacial and cervical myofunctional intervention

Reason 2. Studies involving adults with a history of psychiatric, neurological, neuromuscular, neurodegenerative, or oncological disorders in the orofacial and/or cervical region

Reason 3. Studies involving adults with cleft lip and palate, oropharyngeal dysphagia, obstructive sleep apnea syndrome, temporomandibular joint disorder, and/or facial trauma

Reason 4. Secondary studies, such as abstracts, systematic reviews, narrative reviews, integrative reviews, meta-analyses, letters to the editor, guidelines, and clinical practice guidelines

Reason 5. Studies with replicate samples

Source: Page et al.^(25)^. For more information, visit PRISMA^(26)^ website

Finally, a narrative synthesis described the orofacial and cervical myofunctional intervention programs included in this study to present postoperative therapy organization, composition, and objectives.

## RESULTS

### Selection of sources of evidence

**Figure 2:**
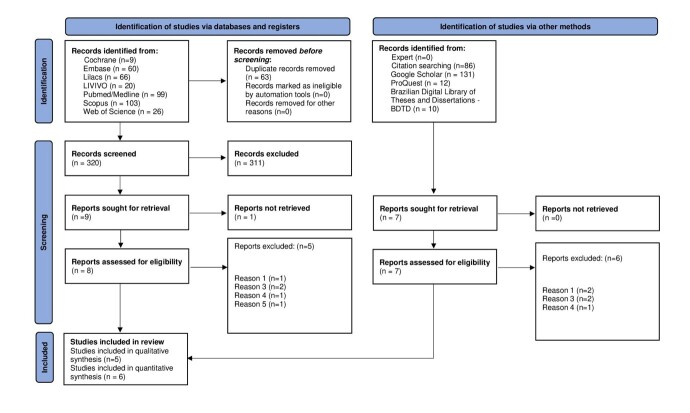
Flowchart of study selection/source of evidence

Altogether, 622 articles were retrieved from databases and gray literature. After removing duplicates, 559 citations were identified. Based on title and abstract analysis, 543 articles were excluded, resulting in the identification of 16 articles, which were retrieved and assessed for eligibility. One of the studies was not retrieved in full. Also, 10 were excluded, with the reasons for exclusion and their respective references given in Appendix B. Thus, five studies^(5,11,18-20)^ were considered eligible for inclusion in this scoping review, as illustrated in Figure 2.

### Characteristics of sources of evidence

The five eligible studies^(5,11,18-20)^ were developed in Brazil and published in English between 2006 and 2018. Participants' ages ranged from 19 to 31 years, with an average proportion of 60% females and 40% males. The studies divided the samples into intervention and control groups, employing a randomized clinical^(5,11)^ or cohort^(18-20)^ design. The sample size ranged from 10 to 15 participants, with follow-up periods of 15 days to 6 months.

### Results of individual sources of evidence

Table 1 describes the authors, country, year of publication, methodological designs, evaluation instruments or technologies, and intervention characteristics, whether through intervention programs or myofunctional exercises of the five eligible studies, also summarizing the intervention outcomes.

Assessment techniques included electromyography (EMG)^(11,20)^, ultrasound^(18,20)^, validated protocols such as the Orofacial Myofunctional Assessment Protocol (MBGR)^(5)^ and the Orofacial Myofunctional Evaluation Protocol with Scores-extended (OMES-E)^(11)^, and the self-reported Oral Health Impact Profile (OHIP-14)^(5)^.

In addition to the validated protocols, the assessments investigated the following objects: the predominant mode of respiratory function (mouth, nasal, or mixed), the oral phase of swallowing, chewing, speech, and aspects of lip and tongue tonicity and posture^(18)^.

Evaluations were conducted after orthognathic surgery, pre- and post-orofacial and cervical myofunctional intervention. However, the interval varied between 3 months^(5,11)^, 6 months^(11)^, and from 3 years to 3 years and 8 months^(19,20)^ after the intervention. Furthermore, one study conducted preoperative assessment, with an average of 3 months before the surgical intervention, followed by analysis performed 6 to 8 months after orthognathic surgery^(18)^. The studies also varied in the number and frequency of sessions, from a weekly frequency over 8 to 15 weeks^(5,11)^ to an extended period of 6 months^(18)^ or 11 months^(19,20)^.

Regarding the intervention process, three studies^(18-20)^ addressed post-surgical guidelines, isometric, isotonic, postural, and functional exercises, and tactile and thermal sensory stimulation. The remaining two studies^(5,11)^ incorporated isometric and isotonic exercises with tactile-kinesthetic and thermal stimulation to highlight their contributions to the stomatognathic system’s functional improvement.

Speech-language-hearing intervention aimed to verify the effect of OMT on orofacial functions and quality of life of individuals undergoing orthognathic surgery^(5)^. It applied tactile-kinesthetic and thermal stimulation in the lower face; isotonic exercises in lips, tongue, and jaw; exercises to adjust the tone of tongue, lips, cheeks, and chin; and exercises to optimize the morphological aspects of the lips, considering the frequency of shortened upper lip or everted lip.

The functional training approach^(5)^ was designed to improve the habitual posture of the lips, jaw, and tongue, and optimize orofacial functions, such as breathing (by stimulating nasal breathing and training the lower middle respiratory tract), chewing (by implementing the alternating or simultaneous bilateral chewing pattern), swallowing (by adjusting lip and tongue tone and mobility), and speech (phonetic origin) (by adjusting tongue mobility, tone, and proprioception and the articulatory pattern). Exercises were also used to aid expressiveness during oral communication to maintain orofacial and functional aesthetic balance.

The Post Orthognathic Surgery Therapy Protocol^(11)^ is a structured plan developed to analyze the clinical and electromyographic aspects of masticatory function 3 and 6 months after orthognathic surgery. It includes the following elements: tactile and thermal stimulation activities are conducted in the first two sessions, associated with swallowing proprioception and isotonic exercises; the latter two continue until the eighth and third sessions, respectively. In the second session, exercises aimed at developing muscle tone, correcting habitual tongue posture, and training the simultaneous bilateral masticatory pattern are introduced, remaining until the eighth, fourth, and third sessions, respectively. Training the alternating bilateral masticatory pattern and the phonetic approach to speech are incorporated in the fourth session and continue until the eighth session. Therapeutic progress is reassessed in the ninth session, and participants receive final instructions in the tenth session.

A structured plan was developed to determine the influence of interdisciplinary treatment in Class III patients and bilateral changes in the thickness of the masseter muscle at rest and when biting^(18)^. It also aimed to analyze the impact of interdisciplinary treatment involving orthodontics, surgery, and OMT in patients with Class III DFD on the electromyographic activity of the masticatory muscles^(20)^ and on the thickness of the masseter muscle^(19)^ after 3 years of surgical correction.

Therefore, post-surgical OMT^(18-20)^ consisted of the following interventions: application of warm compresses and massage starting 2 weeks after surgery to reduce facial edema, relax the jaw elevator muscles, and improve jaw movement. Guidelines regarding food consistency were provided, starting with a liquid diet and gradually progressing to a solid diet. Patients reporting temporomandibular joint (TMJ) pain or discomfort were recommended to maintain a soft diet and practice simultaneous bilateral chewing. Furthermore, specific exercises were prescribed to promote the gradual recovery of jaw movement, including techniques to strengthen and improve lip and tongue posture. When changes in swallowing, chewing, or speech were identified, they performed corrective exercises targeted at these specific aspects for each patient’s comprehensive and personalized rehabilitation^(18-20)^. Tactile and thermal sensory stimulation was also applied as part of the rehabilitation process^(18-20)^.

It is essential to emphasize that the masticatory function was practiced with natural foods throughout the intervention sessions, with a gradual progression of food consistency during the postoperative period. Functional training focused on stretching the jaw elevator and masseter muscles to restore mandibular opening, laterality, and protrusion movements, often compromised by mandibular orthognathic surgery^(19)^.

### Summary of results

The results of the evaluation process were presented in a bubble plot (Figure 1), providing a clear and comparative visualization of the data. Note that most studies had positive results, indicated by green bubbles, representing improvements in the SS structural patterns and functioning after the orthognathic surgery. Studies with negative results, indicated by red bubbles, showed worsening patterns. The bubble size reflects each study’s sample size, allowing for a comparative analysis of OMT effectiveness.

Thus, specific exercise regimens were developed to improve SS functional patterns after the orthognathic surgery. Within the scope of this study, positive results were identified that corroborate the effectiveness of the proposed interventions, including a substantial increase in the thickness of the right and left temporal muscles^(20)^ and right and left masseter^(18,19)^ at rest and when biting^(18)^; significant improvement in the performance of orofacial functions^(5)^, such as breathing, chewing, swallowing, and speech, suggesting optimized SS functioning; an increase in the OHIP-14 score^(5)^ and a maximum score in the Oral Health Assessment (OMS-E)^(11)^, indicating a better perception of oral health by the participants after the intervention; and improved lower lip tone and tongue mobility and more masticatory cycles^(11)^.

As neutral results, we highlight the absence of changes in head movements and food spillage^(11)^, differences in the thickness of the masseter muscle between the sides^(18)^, and statistical differences for the left masseter in contraction when biting^(19)^. Despite the numerical increase in individuals who achieved adequate lip mobility, the differences were not statistically significant^(11)^.

As negative results, we highlight the lack of progress in the chewing, swallowing, and speech parameters in the group that did not undergo OMT^(5)^; lack of difference in bite and individuals with abnormal head posture in the group that underwent OMT^(11)^; lack of statistical significance for bite, high number of participants with changes in lower lip tone and lip and tongue mobility in the group that did not undergo OMT^(11)^.

## DISCUSSION

This scoping review aimed to map the evidence on orofacial and cervical myofunctional intervention programs in adults with DFD undergoing orthognathic surgery. The importance of this topic lies in the need to understand the functional and structural outcomes of orofacial myofunctional therapy in the rehabilitation of patients undergoing orthognathic surgery.

The sample profile characterization regarding predominant sex and age can be justified by the craniofacial development and growth process. Craniofacial skeletal characteristics are defined during puberty. However, it is in adulthood that DFD, consolidated by the completion of craniofacial growth^(21)^, determines adaptive functional patterns regarding habitual oral posture, breathing, chewing, swallowing, and speech articulation points, compatible with one’s anatomical characteristics. Sex is an intervening factor in skeletal development, given that females tend to reach their adult facial shape earlier than males^(22)^. Furthermore, DFD may result in a greater propensity for orofacial pain in females than in males, accompanied by a heightened concern with aesthetic and social aspects^(23)^.

Intervention studies are primary investigations that involve the controlled and intentional manipulation of an exposure factor to analyze the effects of promoted changes. These studies have a prospective design and are often used in randomized controlled clinical trials^(24)^. This review selected therapeutic studies to ensure comparability across multiple variables and reduce the risk of selection and confounding bias.

Thus, the review focused on mapping and describing the reported results of orofacial and cervical myofunctional intervention programs and myofunctional exercises on orofacial functions and structures in patients undergoing orthognathic surgery. In addition to the clinical trials, three cohort studies were included^(18-20)^, a design that contributes to the understanding of long-term effects and the identification of causal associations in contexts where clinical trials may not be feasible or ethical. However, a limitation associated with cohort studies is their reduced ability to establish direct causality, compared with randomized clinical trials, due to the possibility of residual confounding factors^(24)^.

In eligible studies, orofacial structures and functions were assessed with instrumental methods such as surface EMG^(11,20)^ and ultrasound^(18,19)^. These methods provide a quantitative and complementary approach essential for the clinical diagnosis of orofacial myofunctional disorders. Ultrasound is used as a morphometric technique to measure orofacial muscle thickness and monitor tongue movements during chewing, swallowing, and speech. In contrast, surface EMG is an electrophysiological instrument used to capture the electrical activity of orofacial and cervical muscles at rest, maximal isometric contraction, and specific orofacial function tasks.

In addition to instrumental assessments, orofacial myofunctional evaluation used validated instruments – namely, MBGR^(5)^ and OMES-E^(11)^. Applying these assessment protocols is essential for a detailed and comprehensive orofacial myofunctional assessment of SS anatomical and functional conditions.

Instrumental assessments offer a clear understanding of the anatomy and physiology of the systems involved and provide biofeedback on muscle activity. Nonetheless, it is important to recognize that the high cost of these technologies can limit their accessibility and widespread use. Furthermore, effective treatment planning requires a thorough and comprehensive clinical assessment and orofacial myofunctional evaluation. Thus, the combination of clinical and instrumental assessments allows for a more precise and personalized therapeutic approach, which can optimize therapeutic outcomes and patients' quality of life.

The orofacial and cervical myofunctional intervention developed for individuals with DFD undergoing orthognathic surgery consists of post-surgical guidelines, tactile and thermal sensory stimulation, isometric, isotonic, postural, and functional exercises^(18-20)^, and isometric and isotonic exercises associated with tactile-kinesthetic and thermal stimulation^(5,11)^. Hence, this study aimed to map the synthesis of evidence on orofacial and cervical myofunctional intervention programs developed for adults undergoing orthognathic surgery and their influence on the SS.

Speech-language-hearing pathologists play a crucial role in interdisciplinary orthognathic surgery teams, especially in the assessment of the orofacial myofunctional system before and after surgery^(21)^. The preoperative orofacial myofunctional assessment, performed by the five eligible studies in this review^(5,11,18-20)^, aims to identify and characterize muscular and functional changes to develop specific intervention protocols, with guidelines and exercises targeted at the identified orofacial myofunctional disorders, and to prepare the patient for surgery and assist in postoperative recovery. Furthermore, the preoperative assessment provides the team and the patient with a comparative factor between the assessments and periods analyzed to verify SS characteristics, compensations, and adaptations resulting from DFD^(1,21,22)^.

The compression of SS anatomical and functional conditions requires the continuity of speech-language-hearing interventions before and after the surgery. Interventions are recommended 30 to 60 days before surgery to provide guidance and clarify the perception of appropriate muscle mechanisms and patterns at rest and during the performance of orofacial functions^(21,22)^. This also helps develop a new proprioceptive system and optimizes the SS functional conditions in preparation for anatomical changes following surgery.

The postsurgical period begins within the first 24 hours or approximately 20 to 60 days after the procedure^(7,21,22)^. This phase is characterized by a multifaceted approach and includes strategies to reduce facial edema, rehabilitate orofacial sensitivity, stimulate facial movements, and increase the range of mandibular movements^(7,21)^. Similarly, a gradual protocol for reintroducing food consistencies is established, as well as adjustments and adaptations of orofacial functions, aiming to achieve effective SS restoration. Thus, the beginning of the intervention in the studies included in this review is aligned with the period recommended in the literature. The intervention programs described in the eligible studies consist of isometric, isotonic, postural, and functional exercises, and tactile-kinesthetic and thermal stimuli. These techniques aim to enhance treatment efficacy and facilitate comprehensive recovery.

A study^(19)^ included in this review evaluated the impact of myofunctional intervention on the thickness of the left masseter muscle during contraction in 28 participants, 13 of whom underwent an interdisciplinary protocol (orthodontics, surgery, and OMT) for Class III DFD. After 3 years, the results showed that the thickness of the left masseter muscle during contraction in the treated patients did not differ significantly from that observed in individuals without skeletal changes, indicating an approximation to the normal pattern. Although no significant increase in muscle thickness was observed, the treatment harmonized thickness at rest between the sides and established favorable conditions for dental occlusion and muscle flexibility. The findings reinforce the importance of integrated therapy for functional and aesthetic rehabilitation in patients with Class III deformity.

Therefore, combining therapeutic strategies and adhering to intervention protocols are crucial for optimizing postoperative outcomes. The integration of these practices highlights the need for systematic, personalized, and individualized management during the postoperative period, reflecting an evidence-based care model for orofacial rehabilitation, and ensuring maximum functional and aesthetic recovery for the patient.

### Limitations

The extensive literature search for articles on this topic, encompassing international and multidisciplinary databases, strictly followed the scoping review guidelines. Data synthesis and gray literature survey are clear strengths of this study, along with the inclusion of studies involving SS functions, speech-language-hearing therapy, and orthognathic surgery interventions.

The most evident limitation is the substantial gap in findings, along with the variability of intervention programs and the lack of validated protocols for clinical orofacial myofunctional assessment and instrumental assessment of orofacial structures and functions in patients undergoing orthognathic surgery. This gap limits the generalizability of the results and the practical application of the findings.

Moreover, the studies provide little detail of the exercises used in the interventions, with a focus predominantly on general objectives and without adequate specification of the necessary number of sessions and repetitions to guarantee effective therapeutic efficacy.

Furthermore, these studies used heterogeneous methods, such as differences in study designs, small sample sizes, and variations in the evaluation criteria for myofunctional interventions. This diversity makes it difficult to directly compare results and draw robust conclusions. Furthermore, most studies lack long-term follow-up, which limits understanding of the sustained effects of the proposed interventions.

Finally, it is important to emphasize that scoping reviews, unlike systematic reviews, do not require a critical assessment of the studies’ methodological quality. Therefore, the limitations inherent to the interpretation of results must be recognized and considered.

### Suggestions for future research

Based on the reviewed literature, it is possible to understand why primary studies have defined specific objectives and exercise regimens to improve the SE of patients with dentofacial deformities undergoing orthognathic surgery. However, there is still a need for more specific, detailed, and integrated analysis. Therefore, if primary studies continue to be developed in the same way, a definitive answer remains elusive. Therefore, based on our findings, we propose the following directions for future research and considerations for clinical practice.

Hence, it is imperative to validate an orofacial myofunctional assessment instrument for pre- and postoperative use in patients undergoing orthognathic surgery to address these gaps and improve clinical practice. This instrument should be developed to establish clear assessment standards, characterize orofacial myofunctional disorders in detail, and enable the formulation of individualized, evidence-based treatment plans.

It is also recommended that an orofacial and cervical myofunctional intervention program be developed for adults undergoing orthognathic surgery. This program should define the role of speech-language-hearing pathologists in interdisciplinary teams, the intervention periods, and specific therapeutic objectives, and identify the parameters to be assessed. Implementing a structured, evidence-based program will provide robust practical guidelines, guiding clinical practice regarding intervention duration, assessed parameters, and goals to be achieved.

These strategies aim to improve the effectiveness of myofunctional interventions, promoting more precise and evidence-based therapeutic planning, thus contributing to a more effective SS postoperative recovery with fewer recurrences.

## CONCLUSION

The evidence syntheses identified in the mapping indicate that orofacial and cervical myofunctional intervention programs developed for adults with DDF undergoing orthognathic surgery help to recover and improve SS functioning.

Eligible studies, which include both randomized clinical designs and cohort studies, demonstrate that structured interventions consisting of isometric, isotonic, postural, and functional exercises result in substantial improvements in masseter muscle thickness, orofacial function performance, and overall perception of oral health.

Thus, orofacial myofunctional interventions are fundamental in postoperative rehabilitation, as they contribute to optimizing SS functioning, although the variability in results indicates the need for individualized approaches and well-founded and validated assessments.

## Appendix A Search strategies in databases and gray literature

**Table:**

**Database**	**Search** (October 25, 2023)
**Embase**	#1 'myofunctional therapy'/exp OR 'myofunctional therapy' OR 'myofunctional therapies' OR 'orofacial myotherapy' OR 'oral myotherapy' OR 'orofacial myology' OR 'oral exercise' OR 'promotion program' OR 'long-term care prevention programs' OR 'resistance training'/exp OR 'resistance training' OR 'strength training'/exp OR 'strength training' OR 'exercise program'/exp OR 'exercise program' OR 'exercise therapy'/exp OR 'exercise therapy' OR 'remedial exercise' OR 'remedial exercises' OR 'exercise therapies' OR 'rehabilitation exercise' OR 'rehabilitation'/exp OR 'rehabilitation' OR 'habilitation' OR 'speech therapy'/exp OR 'speech therapy' OR 'speech therapies' #2 'dentofacial deformities' OR 'dentofacial deformity' OR 'dentofacial abnormalities' OR 'dentofacial abnormality' OR 'orthognathic surgery' OR 'orthognathic surgeries' OR 'orthognathic surgical procedures' OR 'orthognathic surgical procedure' OR 'maxillo-mandibular surgery' OR 'maxillo mandibular surgery' OR 'maxillofacial orthognathic surgery' OR 'maxillofacial orthognathic surgeries' OR 'surgical-orthodontic' OR 'orthognathic' OR 'orthodontic-surgical' OR 'orthodontic-surgery' OR 'jaw surgeries' OR 'jaw surgery' #3 'stomatognathic system' OR 'stomatognathic systems' OR 'masticatory system' OR 'masticatory systems' OR 'mastication' OR 'chewing' OR 'sucking behavior' OR 'sucking behaviors' OR 'sucking' OR 'deglutition' OR 'deglutitions' OR 'swallowing' OR 'swallowings' OR 'speech' OR 'public speaking' OR 'respiration' OR 'breathing' #4 #1 AND #2 AND #3 #5 #4 AND [embase]/lim NOT ([embase]/lim AND [medline]/lim)
**LILACS**	(“myofunctional therapy” OR “myofunctional therapies” OR “orofacial myotherapy” OR “oral myotherapy” OR “orofacial myology” OR “oral exercise” OR “promotion program” OR “long-term care prevention programs” OR “resistance training” OR “strength training” OR “exercise program” OR “exercise therapy” OR “remedial exercise” OR “remedial exercises” OR “exercise therapies” OR “rehabilitation exercise” OR “rehabilitation” OR “habilitation” OR “speech therapy” OR “speech therapies” OR “terapia miofuncional” OR “miologia orofacial” OR “treinamento de força” OR “treino de força” OR “treino por exercício” OR “exercício de reabilitação” OR “exercício terapêutico” OR “reabilitação” OR “habilitação” OR “reabilitação da fala” OR “reeducação da fala” OR “treinamento de fala” OR “terapia miofuncional” OR “entrenamiento de fuerza” OR “terapia por ejercicio” OR “rehabilitación” OR “logopedia”) AND (“Dentofacial Deformities” OR “Dentofacial Deformity” OR “Dentofacial Abnormalities” OR “Dentofacial Abnormality” OR “Orthognathic Surgery” OR “Orthognathic Surgery” OR “Orthognathic Surgeries” OR “Maxillofacial Orthognathic Surgery” OR “Orthognathic Surgical Procedures” OR “Orthognathic Surgical Procedure” OR “Maxillo-Mandibular Surgery” OR “Maxillo Mandibular Surgery” OR “Maxillofacial Orthognathic Surgery” OR “Maxillofacial Orthognathic Surgeries” OR “surgical-orthodontic” OR “Orthognatic” OR “orthodontic-surgical” OR “orthodontic-surgery” OR “jaw surgeries” OR “jaw surgery” OR “deformidades dentofaciais” OR “cirurgia ortognótica” OR “procedimentos cirúrgicos ortognáticos” OR “cirurgia de mandíbula” OR “cirurgia mandibular” OR “cirurgia maxilomandibular” OR “cirurgia maxilomandibulares” OR “cirurgias ortognática maxilofaciais” OR “deformidades dentofaciales” OR “procedimentos quirúrgicos ortognáticos”) AND (“Stomatognathic System” OR “Stomatognathic Systems” OR “Masticatory System” OR “Masticatory Systems” OR “Mastication” OR “Chewing” OR “Sucking Behavior” OR “Sucking Behavior” OR “sucking” OR “Deglutition” OR “Deglutitions” OR “Swallowing” OR “Swallowings” OR “Speech” OR “Public Speaking” OR “Respiration” OR “Breathing” OR “sistema estomatognático” OR “sistema mastigatório” OR “mastigação” OR “Comportamento de Sucção” OR “Conducta en la Lactancia” OR “deglutição” OR “fala” OR “respiração” OR “sistema estomatognático” OR “sistema masticatorio” OR “masticación” OR “conducta en la lactancia” OR “deglución” OR “habla” OR “respiración”)
**PubMed/ Medline**	(“myofunctional therapy”[Mesh] OR “myofunctional therapy” OR “myofunctional therapies” OR “orofacial myotherapy” OR “oral myotherapy” OR “orofacial myology” OR “oral exercise” OR “promotion program” OR “long-term care prevention programs” OR “resistance training”[Mesh] OR “strength training” OR “exercise program” OR “exercise therapy”[Mesh] OR “remedial exercise” OR “rehabilitation exercise” OR “rehabilitation”[Mesh] OR “habilitation” OR “speech therapy” OR “speech therapies”) AND (“Dentofacial Deformities”[Mesh] OR “Dentofacial Deformities” OR “Dentofacial Deformity” OR “Dentofacial Abnormalities” OR “Dentofacial Abnormality” OR “Orthognathic Surgery”[Mesh] OR “Orthognathic Surgery” OR “Orthognathic Surgeries” OR “Maxillofacial Orthognathic Surgery” OR “Orthognathic Surgical Procedures” OR “Orthognathic Surgical Procedures”[Mesh] OR “Orthognathic Surgical Procedures” OR “Orthognathic Surgical Procedure” OR “Maxillo-Mandibular Surgery” OR “Maxillo Mandibular Surgery” OR “Maxillofacial Orthognathic Surgery” OR “Maxillofacial Orthognathic Surgeries” OR “surgical-orthodontic” OR “Orthognatic” OR “orthodontic-surgical” OR “orthodontic-surgery”) AND (“Stomatognathic System”[MeSH] OR “Stomatognathic System” OR “Stomatognathic Systems” OR “Masticatory System” OR “Masticatory Systems” OR “Mastication”[MesH] OR “Mastication” OR “Chewing” OR “Sucking Behavior”[Mesh] OR “Sucking Behavior” OR “Sucking Behaviors” OR “sucking” OR “Deglutition”[Mesh] OR “Deglutition” OR “Deglutitions” OR “Swallowing” OR “Swallowings” OR “Speech”[Mesh] OR “Speech” OR “Public Speaking” OR “Respiration”[Mesh] OR “Breathing”)
**Scopus**	(TITLE-ABS-KEY (“myofunctional therapy” OR “myofunctional therapies” OR “orofacial myotherapy” OR “oral myotherapy” OR “orofacial myology” OR “oral exercise” OR “promotion program” OR “long-term care prevention programs” OR “resistance training” OR “strength training” OR “exercise program” OR “exercise therapy” OR “remedial exercise” OR “rehabilitation exercise” OR “rehabilitation” OR “habilitation” OR “speech therapy” OR “speech therapies”) AND TITLE-ABS-KEY (“Dentofacial Deformities” OR “Dentofacial Deformity” OR “Dentofacial Abnormalities” OR “Dentofacial Abnormality” OR “Orthognathic Surgery” OR “Orthognathic Surgeries” OR “Maxillofacial Orthognathic Surgery” OR “Orthognathic Surgical Procedures” OR “Orthognathic Surgical Procedure” OR “Maxillo-Mandibular Surgery” OR “Maxillo Mandibular Surgery” OR “Maxillofacial Orthognathic Surgery” OR “Maxillofacial Orthognathic Surgeries” OR “surgical-orthodontic” OR “Orthognatic” OR “orthodontic-surgical” OR “orthodontic-surgery”) AND TITLE-ABS-KEY (“Stomatognathic System” OR “Stomatognathic Systems” OR “Masticatory System” OR “Masticatory Systems” OR “Mastication” OR “Chewing” OR “Sucking Behavior” OR “Sucking Behavior’” OR “sucking” OR “Deglutition” OR “Deglutitions” OR “Swallowing” OR “Swallowings” OR “Speech” OR “Public Speaking” OR “Respiration” OR “Breathing”)
**Web of Science**	(“myofunctional therapy” OR “myofunctional therapies” OR “orofacial myotherapy” OR “oral myotherapy” OR “orofacial myology” OR “oral exercise” OR “promotion program” OR “long-term care prevention programs” OR “resistance training” OR “strength training” OR “exercise program” OR “exercise therapy” OR “remedial exercise” OR “rehabilitation exercise” OR “rehabilitation” OR “habilitation” OR “speech therapy” OR “speech therapies”) (Tópico) AND (“Dentofacial Deformities” OR “Dentofacial Deformity” OR “Dentofacial Abnormalities” OR “Dentofacial Abnormality” OR “Orthognathic Surgery” OR “Orthognathic Surgeries” OR “Maxillofacial Orthognathic Surgery” OR “Orthognathic Surgical Procedures” OR “Orthognathic Surgical Procedure” OR “Maxillo-Mandibular Surgery” OR “Maxillo Mandibular Surgery” OR “Maxillofacial Orthognathic Surgery” OR “Maxillofacial Orthognathic Surgeries” OR “surgical-orthodontic” OR “orthognathic” OR “orthodontic-surgical” OR “orthodontic-surgery”) (Tópico) AND (“Stomatognathic System” OR “Stomatognathic Systems” OR “Masticatory System” OR “Masticatory Systems” OR “Mastication” OR “Chewing” OR “Sucking Behavior” OR “Sucking Behavior” OR “sucking” OR “Deglutition” OR “deglutition” OR “swallowing” OR “Swallowings” OR “Speech” OR “Public Speaking” OR “Respiration” OR “Breathing”)
**Cochrane**	(“myofunctional therapy” OR “myofunctional therapies” OR “orofacial myotherapy” OR “oral myotherapy” OR “orofacial myology” OR “oral exercise” OR “promotion program” OR “long-term care prevention programs” OR “resistance training” OR “strength training” OR “exercise program” OR “exercise therapy” OR “remedial exercise” OR “rehabilitation exercise” OR “rehabilitation” OR “habilitation” OR “speech therapy” OR “speech therapies”) in Title Abstract Keyword AND (“Dentofacial Deformities” OR “Dentofacial Deformity” OR “Dentofacial Abnormalities” OR “Dentofacial Abnormality” OR “Orthognathic Surgery” OR “Orthognathic Surgeries” OR “Maxillofacial Orthognathic Surgery” OR “Orthognathic Surgical Procedures” OR “Orthognathic Surgical Procedure” OR “Maxillo-Mandibular Surgery” OR “Maxillo Mandibular Surgery” OR “Maxillofacial Orthognathic Surgery” OR “Maxillofacial Orthognathic Surgeries” OR “surgical-orthodontic” OR “orthognathic” OR “orthodontic-surgical” OR “orthodontic-surgery”) in Title Abstract Keyword AND (“Stomatognathic System” OR “Stomatognathic Systems” OR “Masticatory System” OR “Masticatory Systems” OR “Mastication” OR “Chewing” OR “Sucking Behavior” OR “Sucking Behavior” OR “sucking” OR “Deglutition” OR “deglutition” OR “Swallowing” OR “swallowing” OR “Speech” OR “Public Speaking” OR “Respiration” OR “Breathing”)
**LIVIVO**	(“myofunctional therapy” OR “myofunctional therapies” OR “orofacial myotherapy” OR “oral myotherapy” OR “orofacial myology” OR “oral exercise” OR “promotion program” OR “long-term care prevention programs” OR “resistance training” OR “strength training” OR “exercise program” OR “exercise therapy” OR “remedial exercise” OR “rehabilitation exercise” OR “rehabilitation” OR “habilitation” OR “speech therapy” OR “speech therapies”) AND (“Dentofacial Deformities” OR “Dentofacial Deformity” OR “Dentofacial Abnormalities” OR “Dentofacial Abnormality” OR “Orthognathic Surgery” OR “Orthognathic Surgeries” OR “Maxillofacial Orthognathic Surgery” OR “Orthognathic Surgical Procedures” OR “Orthognathic Surgical Procedure” OR “Maxillo-Mandibular Surgery” OR “Maxillo Mandibular Surgery” OR “Maxillofacial Orthognathic Surgery” OR “Maxillofacial Orthognathic Surgeries” OR “surgical-orthodontic” OR “orthognathic” OR “orthodontic-surgical” OR “orthodontic-surgery”) AND (“Stomatognathic System” OR “Stomatognathic Systems” OR “Masticatory System” OR “Masticatory Systems” OR “Mastication” OR “Chewing” OR “Sucking Behavior” OR “Sucking Behavior” OR “sucking” OR “Deglutition” OR “deglutition” OR “swallowing” OR “Swallowings” OR “Speech” OR “Public Speaking” OR “Respiration” OR “Breathing”)
**Google Scholar**	(“myofunctional therapy” OR “exercise therapy”) AND (“dentofacial deformities” OR “orthognathic surgery”) filetype:PDF
**ProQuest**	NOFT (“myofunctional therapy” OR “myofunctional therapies” OR “orofacial myotherapy” OR “oral myotherapy” OR “orofacial myology” OR “oral exercise” OR “promotion program” OR “long-term care prevention programs” OR “resistance training” OR “strength training” OR “exercise program” OR “exercise therapy” OR “remedial exercise” OR “rehabilitation exercise” OR “rehabilitation” OR “habilitation” OR “speech therapy” OR “speech therapies”) AND NOFT (“Dentofacial Deformities” OR “Dentofacial Deformity” OR “Dentofacial Abnormalities” OR “Dentofacial Abnormality” OR “Orthognathic Surgery” OR “Orthognathic Surgeries” OR “Maxillofacial Orthognathic Surgery” OR “Orthognathic Surgical Procedures” OR “Orthognathic Surgical Procedure” OR “Maxillo-Mandibular Surgery” OR “Maxillo Mandibular Surgery” OR “Maxillofacial Orthognathic Surgery” OR “Maxillofacial Orthognathic Surgeries” OR “surgical-orthodontic” OR “orthognathic” OR “orthodontic-surgical” OR “orthodontic-surgery”) AND NOFT (“Stomatognathic System” OR “Stomatognathic Systems” OR “Masticatory System” OR “Masticatory Systems” OR “Mastication” OR “Chewing” OR “Sucking Behavior” OR “Sucking Behavior” OR “sucking” OR “Deglutition” OR “deglutition” OR “swallowing” OR “Swallowings” OR “Speech” OR “Public Speaking” OR “Respiration” OR “Breathing”)
**Brazilian Digital Library of Theses and Dissertations (BDTD)**	(“myofunctional therapy” OR “myofunctional therapies” OR “orofacial myotherapy” OR “oral myotherapy” OR “orofacial myology” OR “oral exercise” OR “promotion program” OR “long-term care prevention programs” OR “resistance training” OR “strength training” OR “exercise program” OR “exercise therapy” OR “remedial exercise” OR “rehabilitation exercise” OR “rehabilitation” OR “habilitation” OR “speech therapy” OR “speech therapies”) AND (“Dentofacial Deformities” OR “Dentofacial Deformity” OR “Dentofacial Abnormalities” OR “Dentofacial Abnormality” OR “Orthognathic Surgery” OR “Orthognathic Surgeries” OR “Maxillofacial Orthognathic Surgery” OR “Orthognathic Surgical Procedures” OR “Orthognathic Surgical Procedure” OR “Maxillo-Mandibular Surgery” OR “Maxillo Mandibular Surgery” OR “Maxillofacial Orthognathic Surgery” OR “Maxillofacial Orthognathic Surgeries” OR “surgical-orthodontic” OR “orthognathic” OR “orthodontic-surgical” OR “orthodontic-surgery”) AND (“Stomatognathic System” OR “Stomatognathic Systems” OR “Masticatory System” OR “Masticatory Systems” OR “Mastication” OR “Chewing” OR “Sucking Behavior” OR “Sucking Behavior” OR “sucking” OR “Deglutition” OR “deglutition” OR “swallowing” OR “Swallowings” OR “Speech” OR “Public Speaking” OR “Respiration” OR “Breathing”)

## Appendix B Excluded articles and reason for exclusion (n = 10)

**Table:**

**Author**	**Reasons for exclusion**
Altmann^1^	4
Costa^2^	3
Gallerano et al.^3^	3
Lichnowska e Kozakiewicz^4^	4
Mangilli^5^	3
Min et al.^6^	1*
Ohba et al.^7^	1*
Trawitzki et al.^8^	5
Tyndor et al.^9^	3
Watanabe et al.^10^	1

**Caption:** 1. Studies in which health professionals other than speech-language-hearing pathologists performed orofacial and cervical myofunctional intervention; 2. Studies with adults with a history of psychiatric, neurological, neuromuscular, neurodegenerative, or oncological disorders in the orofacial and/or cervical region; 3. Studies involving adults with cleft lip and palate, oropharyngeal dysphagia, obstructive sleep apnea syndrome, temporomandibular disorder, and/or facial trauma; 4. Secondary studies, such as abstracts, systematic reviews, narrative reviews, integrative reviews, meta-analyses, letters to the editor, guidelines, and clinical practice guides; 5. Studies with repeated samples

* The present research team contacted the corresponding authors of the studies excluded due to exclusion criterion 1 by email to verify their academic background. Only Watanabe et al.^10^ responded, stating that the team consisted of orthodontists and dental surgeons, not speech-language-hearing pathologists. In the other cases, there was no response to the email
