# Supplementary material for: Orofacial and cervical myofunctional intervention programs for adults undergoing orthognathic surgery: a scoping review
Source: Codas. 2025 Nov 28;37(6):e20240358. doi: 10.1590/2317-1782/e20240358en (PMC12700110; doi:10.1590/2317-1782/e20240358en)
Supplement: Translation

# Translation: Programas de intervenção miofuncional orofacial e cervical para adultos submetidos a cirurgia ortognática: uma revisão de escopo

**DOI:** 10.1590/2317-1782/e20240358pt

**Authors:** Allya Francisca Marques Borges, Ramon Cipriano Pacheco de Araújo, Samara Fernandes da Silva Souza, Aline Xavier Ferraz, Cristiano Miranda de Araújo, Hipólito Virgílio Magalhães, Renata Veiga Andersen Cavalcanti, Karinna Veríssimo Meira Taveira

**Affiliations:** 1 Programa de Pós-graduação em Fonoaudiologia, Universidade Federal do Rio Grande do Norte – UFRN - Natal (RN), Brasil.; 2 Grupo de Pesquisa Estudos em Motricidade Orofacial e Disfagia Orofaríngea, Universidade Federal do Rio Grande do Norte – UFRN - Natal (RN), Brasil.; 3 Programa de Pós-graduação em Distúrbios da Comunicação, Núcleo de Estudos Avançados em Revisão Sistemática e Meta-análise – NARSM, Universidade Tuiuti do Paraná – UTP - Curitiba (PR), Brasil.; 4 Departamento de Fonoaudiologia, Universidade Federal do Rio Grande do Norte – UFRN - Natal (RN), Brasil.; 5 Programa Associado de Pós-graduação em Fonoaudiologia, Núcleo de Estudos Avançados em Revisão Sistemática e Meta-análise – NARSM, Departamento de Morfologia, Centro de Biociências, Universidade Federal do Rio Grande do Norte – UFRN - Natal (RN), Brasil.

**Keywords:** Terapia Miofuncional, Deformidades Dentofaciais, Cirurgia Ortognática, Sistema Estomatognático, Reabilitação

## Abstract

**Objetivo:**

Mapear os programas de intervenção miofuncional orofacial e cervical desenvolvidos para adultos submetidos à cirurgia ortognática.

**Estratégia de pesquisa:**

Busca nas bases de dados Cochrane, EMBASE, LILACS, LIVIVO, PubMed/Medline, Scopus e Web of Science e na literatura cinzenta.

**Critérios de seleção:**

Foram incluídos estudos que abordaram programas terapêuticos, exercícios miofuncionais, e sua influência no sistema estomatognático de adultos submetidos à cirurgia ortognática, sem delimitação de tempo ou idioma, excluindo estudos secundários, população com idade inferior a 18 anos e comorbidades associadas as deformidades dentofaciais.

**Análise dos dados:**

Extração de dados conduzida por duas revisoras e dados coletados compostos por diferentes tópicos, especialmente processo de avaliação, intervenção miofuncional orofacial e cervical e identificação dos resultados da intervenção.

**Resultados:**

Cinco estudos foram considerados elegíveis. Publicados entre 2006 e 2018, com participantes com idade entre 19 e 31 anos, predomínio do sexo feminino e com uso de diretrizes pós-cirúrgicas compostas por exercícios isométricos, isotônicos, posturais e funcionais, bem como estimulação tátil-cinestésica e térmica, com destaque positivo quanto a melhora funcional do sistema estomatognático.

**Conclusão:**

Os estudos incluídos abordaram a aplicação de terapia miofuncional orofacial após a cirurgia ortognática. As intervenções, ainda não validadas, envolveram protocolos específicos de exercícios posturais e funcionais, além de estimulação tátil-cinestésica e térmica. Os resultados demonstraram uma melhoria significativa no desempenho das funções orofaciais, aprimoramento na percepção subjetiva da saúde oral, e avanços no tônus muscular e na mobilidade das estruturas orofaciais. A terapia miofuncional orofacial apresenta-se como abordagem importante na reabilitação do sistema estomatognático no contexto pós-cirúrgico.

## INTRODUÇÃO

A deformidade dento-esquelética ou dentofacial (DDF) refere-se a uma condição que se manifesta por meio da desarmonia entre os elementos dentários e os maxilares, constituindo-se, portanto, na conjunção de uma maloclusão dentária e esquelética^(1)^. A disposição das bases ósseas e dentárias exerce influência sobre os tecidos moles adjacentes, podendo exercer um impacto significativo na qualidade de vida e nas funções orofaciais do indivíduo^(1,2)^.

A restauração do padrão facial, mediante a correção das desproporções mandibulares, maxilares e/ou região mentual, bem como das assimetrias faciais, após o término do período de crescimento e desenvolvimento craniofacial, demanda a realização da cirurgia ortognática associada ao tratamento ortodôntico^(2-4)^.

A combinação da cirurgia ortognática com o tratamento ortodôntico viabiliza uma correção eficaz das assimetrias faciais, promovendo o estabelecimento de um equilíbrio harmônico entre os dentes, os ossos de sustentação e as estruturas faciais adjacentes^(4)^. Contudo, observam-se casos nos quais persistem padrões funcionais alterados, o que pode comprometer negativamente a manutenção do equilíbrio miofuncional orofacial^(5)^. Assim, para alcançar a estabilidade dos resultados do tratamento, deve ocorrer, associado ao tratamento orto-cirúrgico, um equilíbrio funcional, que ocorre mediante abordagem interdisciplinar e estruturação da terapia miofuncional orofacial (TMO)^(5-7)^.

A TMO abrange um conjunto de técnicas fundamentadas em exercícios específicos direcionados aos músculos orofaciais e cervicais e as funções orofaciais, com o objetivo de promover o desenvolvimento da propriocepção, sensibilidade, mobilidade, coordenação e força das funções e estruturas do sistema estomatognático (SE)^(8)^. A abordagem visa principalmente otimizar as funções vitais do SE, incluindo mastigação, deglutição, sucção, fala e respiração^(5,7,9)^.

A cirurgia ortognática desempenha um importante papel na restauração do equilíbrio das estruturas orofaciais em indivíduos com DDF, o que implica em uma reconfiguração do esquema proprioceptivo para que as estruturas moles desempenhem suas funções satisfatoriamente^(7,10,11)^. Nesse contexto, torna-se imprescindível investigar o impacto da TMO nos aspectos estruturais e funcionais do sistema estomatognático antes e após a correção cirúrgica da DDF. A análise detalhada proporcionará percepções valiosas sobre a adaptação dos músculos e das funções orofaciais após a intervenção cirúrgica, contribuindo significativamente para o desenvolvimento de abordagens terapêuticas mais eficazes e personalizadas para indivíduos com DDF submetidos a cirurgia ortognática, bem como na redução de recidivas decorrentes da persistência de padrões funcionais inadequados.

Diante do ponto de vista do equilíbrio funcional e estrutural do SE de indivíduos com DDF, esta revisão de escopo foi realizada para fornecer uma perspectiva mais abrangente do conhecimento relacionado, mapear a literatura existente, examinar como a pesquisa é conduzida neste campo, analisar lacunas de conhecimento e sugerir pesquisas futuras^(12)^. Portanto, esta revisão apresenta o objetivo de mapear a síntese de evidências sobre programas de intervenção miofuncional orofacial e cervical desenvolvidos para adultos submetidos à cirurgia ortognática e sua influência no sistema estomatognático.

## MÉTODO

Esta revisão de escopo foi conduzida em estrita conformidade com as diretrizes delineadas no manual do Instituto Joanna Briggs (JBI)^(13)^. O protocolo e a revisão foram elaborados utilizando o checklist do *Preferential Reporting Items for Systematic Reviews and Meta-Analyses Extension for Scoping Reviews* (PRISMA-ScR)^(14)^, garantindo, assim, um processo transparente. O protocolo deste estudo foi registrado na plataforma de registro Open Science Framework^(15)^.

### Critérios de elegibilidade

Para considerar a elegibilidade dos estudos a serem incluídos ou excluídos desta revisão, foi utilizado o acrônimo “PCC” (Participantes, Conceito, Contexto): População (P): Adultos com deformidade dentofacial; Conceito (C): Terapia miofuncional orofacial; Contexto (C): Cirurgia ortognática.

### Critérios de inclusão

Foram incluídos nesta revisão, estudos que descreveram, na íntegra, os programas de intervenção miofuncional orofacial e cervical desenvolvidos para adultos submetidos à cirurgia ortognática; estudos que analisaram as ações e os exercícios propostos, assim como seu impacto na mastigação, deglutição, sucção, fala, respiração e na musculatura facial de adultos com deformidades dentofaciais submetidos à cirurgia ortognática; estudos que contemplaram a forma de avaliação, pré e pós intervenção, das funções orofaciais; estudos que relataram o período cirúrgico (pré-cirúrgico, pós-cirúrgico imediato ou pós-cirúrgico tardio) que os exercícios ou ações foram realizados; e, estudos com população do sexo masculino e feminino com idade maior ou igual a 18 anos, sem delimitação de tempo e idioma.

### Critérios de exclusão

Foram excluídos desta revisão: 1. Estudos em que a intervenção miofuncional orofacial e cervical foi realizada por profissionais de saúde sem formação em Fonoaudiologia. 2. Estudos com adultos com histórico de alteração psiquiátrica, neurológica, neuromuscular ou neurodegenerativa e oncológica na região orofacial e/ou cervical. 3. Estudos que apresentaram adultos com fissura labiopalatina, disfagia orofaríngea, síndrome da apneia obstrutiva do sono, disfunção temporomandibular e/ou trauma de face. 4. Estudos secundários como resumos, revisões sistemáticas, narrativa, integrativa, meta-análise, carta ao editor, guideline e guias de prática clínica. 5. Estudos com amostra repetida.

### Fontes de informações e estratégia de busca

Combinações de palavras e truncamentos foram realizadas e ajustados para cada base de dados eletrônica, incluindo Cochrane, EMBASE, Literatura Latino-Americana e do Caribe em Ciências da Saúde (LILACS), LIVIVO, PubMed/Medline, Scopus e Web of Science. A literatura cinzenta também foi utilizada como fonte de informações por meio da Biblioteca Digital Brasileira de Teses e Dissertações (BDTD), Google Acadêmico, ProQuest Teses e Dissertações. Além disto, foi realizada a busca manual nas referências dos estudos incluídos e consulta a um expert para verificar algum possível artigo não incluído. O levantamento bibliográfico foi realizado em 25 de outubro de 2023, cujas estratégias de busca foram elaboradas por uma revisora experiente (KVMT) e refinadas por meio de discussão em equipe. Diante disso, a estratégia de busca final, para cada base de dados e literatura cinzenta, está demonstrada no Apêndice A. Os resultados da busca foram exportados para o software EndNote® (EndNote® X7 Thomson Reuters, Filadélfia, PA).

### Seleção dos estudos

A leitura de títulos e resumo, bem como a leitura dos textos na íntegra foram realizadas por duas revisoras independentes (AFMB e SFSS) por meio do software Rayyan®️^(16)^. Em caso de dúvidas ou conflito, recorreu-se a uma terceira revisora independente (KVMT).

De forma anterior ao processo de análise de títulos e resumos, ocorreu a realização de uma calibração mediante a seleção de 100 referências iniciais recuperadas da literatura^(17)^. A calibração foi realizada, com o intuito de obter o valor > 0.7 no Coeficiente de Concordância de Kappa e assim iniciar o processo de seleção e extração de dados^(17)^.

### Extração de dados

A extração de dados foi conduzida por duas revisoras (AFMB e SFSS) com consulta adicional a outras duas revisoras (KVMT e RVAC) para esclarecer dúvidas relacionadas às variáveis extraídas, aos delineamentos dos estudos e à classificação dos programas de intervenção miofuncional orofacial e exercícios orofaciais. A categorização dos estudos, apresentada na Tabela 1, contemplou informações gerais sobre os estudos, tais como: título, autor, ano, país, delineamento do estudo, objetivo do estudo, características metodológicas (amostra e idade), avaliação (instrumentos analisados e parâmetros temporais), classificação da deformidade dentofacial, período cirúrgico no qual os exercícios miofuncionais foram realizados, e intervenção (classificação quanto ao tipo de programa ou exercícios propostos e frequência). Além disso, a Tabela 1 apresenta uma síntese dos desfechos observados, destacando os efeitos funcionais e musculares decorrentes das intervenções analisadas. Quando os dados estavam incompletos, foram realizadas três tentativas de contactar os autores (primeiro e último autor, e autor correspondente) para obter estas informações, não havendo resposta, o artigo foi excluído.

**Tabela 1:** Caracterização dos estudos que analisaram o impacto da terapia miofuncional orofacial em voluntários submetidos a cirurgia ortognática (n=5)

**Autor, Ano, País, Desenho de Estudo**	**Objetivo**	**Características metodológicas**		**Avaliação**		**Classificação da deformidade dentofacial**	**Período de cirurgia**	**Intervenção**		**Principais resultados**
		**Amostra**	**Idade**	**Instrumentos utilizados**	**Parâmetros temporais**			**Programas/Exercícios**	**Frequência**	
Migliorucci et al.^(5)^, 2017, Brasil, Ensaio clínico randomizado	Verificar o impacto da terapia miofuncional nas funções orofaciais e qualidade de vida de pacientes submetidos à cirurgia ortognática.	Grupo TMO: 12 (7F, 5M)	± 26,35	Protocolo de Avaliação Miofuncional Orofacial (MBGR) e Perfil do Questionário de Impacto na Saúde Bucal-OHIP-14	Antes do procedimento cirúrgico e 3 meses após a cirurgia ortognática.	Classe II: 5 Classe III: 7	A intervenção teve início 40 dias após a cirurgia.	Estimulação tátil-cinestésica e térmica. Exercícios isométricos, isotônicos e funcionais	Uma vez por semana durante 8 a 15 semanas	Melhora significativa nas funções orofaciais, como respiração (p=0,005), mastigação (p=0,006), deglutição (p=0,003) e fala (p=0,012). Impacto positivo na qualidade de vida, conforme o OHIP-14 (p=0,018)
		Grupo sem TMO: 12(7F, 5M)	± 26,35			Classe II: 5 Classe III: 7		Não completou o programa de exercícios		Melhora significativa na respiração (p=0,044) e na qualidade de vida, conforme protocolo OHIP-14 (p=0,003). Ausência de evolução nos parâmetros de mastigação (p=0,757), deglutição (p=0,060) e fala (p=0,539)
Prado et al.^(11)^, 2018, Brasil, Ensaio clínico randomizado	Investigar os efeitos da terapia miofuncional orofacial na função mastigatória de indivíduos com deformidades dentofacial submetidos à cirurgia ortognática.	GT OMT: 13(7F, 6M)	29.31 (± 8.87)	Protocolo de Avaliação Miofuncional Orofacial com Escores Expandidos (AMIOFE-E) e eletromiografia	Antes do procedimento cirúrgico e após o procedimento cirúrgico (três e seis meses)	Classe II: 4 Classe III: 9	A intervenção começou 30 dias após a cirurgia	Estimulação tátil-cinestésica e térmica. Exercícios isométricos, isotônicos, posturais e funcionais.	1 vez/semana durante 10 semanas.	Aumento na pontuação máxima do AMIOFE-E de P0 (13,23 ± 3,06) para P1 (15,92 ± 3,84) e P2 (16,0 ± 3,51), e de P0 (4,3 ± 2,56) para P2 (6,92 ± 2,78). O número de ciclos mastigatórios cresceu de P0 (11,34 ± 2,87) para P2 (13,79 ± 2,44). O tônus labial aumentou de P0 (0 - 0%) para P1 (6 - 46,15%) e P2 (8 - 61,54%), e a mobilidade de P0 (4 - 30,77%) para P1 e P2 (11 - 84,61%). Não houve diferenças significativas na mordida, movimentos da cabeça, escape alimentar e tônus da língua. A postura de cabeça alterada persistiu em 2 a 8 indivíduos.
		UTG TMO: 10 (7F, 3M)	31.20 (± 7.02)			Classe II: 7 Classe III: 3	A intervenção começou seis meses após a cirurgia, após a conclusão de todas as avaliações iniciais	Durante todo o período de avaliação, o grupo não se envolveu no programa de exercícios. A intervenção ocorreu após a consolidação do estudo.		Ausência de diferenças significativas para a mordida entre os períodos, porém observou-se um maior número de indivíduos com tônus do lábio inferior e mobilidade de lábios e língua alterados.
Trawitzki et al.^(18)^, 2006, Brasil, Coorte	Determinar o efeito do tratamento interdisciplinar em paciente com deformidades dentofacial de Classe III, analisando a atividade eletromiográfica dos músculos temporais e masseter durante a mastigação e mordida.	Grupo P1**: 15 (4M, 11F)	M: ± 21,2 anos F: ± 24,9 anos	Ultrassonografia	3 meses antes da cirurgia ortognática*	Classe III	A intervenção iniciou 15 dias após a cirurgia, com duração de 6 meses de intervenção*	O grupo P1 é idêntico ao grupo P2. Porém, está sendo utilizado como referência para avaliação pré-cirúrgica no grupo P2.		Não há resultados a serem analisados.
		Grupo P2**: 15 (4M, 11F)	M: ± 21,2 anos F: ± 24,9 anos		6 a 8 meses após a cirurgia ortognática*	Classe III corrigida		Orientações pós-cirúrgicas. Exercícios isométricos, isotônicos, posturais e funcionais. Estimulação sensorial tátil e térmica*	Uma vez por semana durante 6 meses.	Aumento significativo da espessura do masseter em P2, tanto em repouso quanto na mordida, em comparação a P1. No entanto, os valores de P2 permaneceram inferiores ao do Grupo Controle, bilateralmente.
		Grupo controle: 15 (4M, 11F)	M: ± 20,8 anos F: ± 24 anos		Antes e depois da cirurgia ortognática, nos períodos mencionados nos grupos experimentais.	Ausência de deformidade dentofacial	Não foi realizado TMO	Não realizou o programa de exercícios.		Valores superiores e com significância estatística, quando comparado à P1 e P2, em relação a espessura do músculo masseter, bilateralmente.
Trawitzki et al.^(19)^, 2011, Brasil, Coorte	Analisar o efeito do tratamento ortodôntico integrado, da cirurgia ortognática e da terapia miofuncional orofacial na espessura do músculo masseter em pacientes com deformidades dentofacial de classe III, três anos após a cirurgia ortognática.	Grupo P1**: 13 (3M, 10F)	M: ± 22 anos F: ± 27 anos	Ultrassonografia	Dois a 74 dias antes da cirurgia ortognática.	Classe III	A intervenção iniciou 15 dias após a cirurgia, com duração de 6 meses de intervenção.	O grupo P1 é idêntico ao grupo P3. Porém, está sendo utilizado como referência para avaliação pré-cirúrgica no grupo P3.		Não há resultados a serem analisados.
		Grupo P3**: 13 (3M, 10F)	M: ± 22 anos F: ± 27 anos		Os dados foram obtidos durante um período de 3 anos a 3 anos e 8 meses pós-operatório (3 anos e 2 meses).	Classe III corrigida		Orientações pós-cirúrgicas. Exercícios isométricos, isotônicos, posturais e funcionais. Estimulação sensorial tátil e térmica*	Uma vez por semana durante os primeiros 11 meses após a cirurgia.	Aumento significativo na espessura do masseter direito e esquerdo (P < 0,01). Em comparação ao Grupo Controle, observou-se diferença significativa para o masseter direito em repouso (P < 0,01) e mordida (P < 0,05), e para o masseter esquerdo em repouso (P < 0,05). Ausência de diferença significativa para o masseter esquerdo na mordida (P = 0,05).
		Grupo controle: 15 (4M, 11F)	M: ± 21 anos F: ± 24 anos		Antes e depois da cirurgia ortognática, nos períodos mencionados nos grupos experimentais.	Ausência de deformidade dentofacial	Não foi realizado TMO.	Não realizou o programa de exercícios.		Não há resultados a serem relatados.
Trawitzki et al.^(20)^, 2010, Brasil, Coorte	Investigar o efeito do tratamento interdisciplinar na atividade eletromiográfica dos músculos mastigatórios em pacientes com deformidades dentofacial de Classe III, três anos após a correção cirúrgica.	Grupo P1***:13(3M, 10F)	M: ± 22 anos F: ± 27 anos	Eletromiografia	Antes do procedimento cirúrgico.	Classe III	A intervenção iniciou 15 dias após a cirurgia, com duração de 6 meses de intervenção.	O grupo P1 é idêntico ao grupo P3. Porém, está sendo utilizado como referência para avaliação pré-cirúrgica no grupo P3.		Nos músculos T e M apresentou diferenças significativas entre o P1 e os Grupos P3, na mastigação e mordida, com valores maiores em P3***
		Grupo P3***:13 (3M, 10F)	M: ± 22 anos F: ± 27 anos		Os dados foram coletados durante um período de 3 anos a 3 anos e 8 meses de pós-operatório (3 anos e 2 meses).	Classe III corrigida		Orientações pós-cirúrgicas. Exercícios isométricos, isotônicos, posturais e funcionais. Estimulação sensorial tátil e térmica*	Durou em média 11 meses.	Na atividade EMG, houve disparidade significativa (P < 0,05) nos valores do músculo M durante a mastigação e a mordida, com valores mais elevados em P3***
		Grupo controle: 15 (11F, 4M)	M: ± 21 anos F: ± 24 anos		Antes e depois da cirurgia ortognática, nos períodos mencionados nos grupos mencionados.	Ausência de deformidade dentofacial.	Não foi realizado TMO.	Não realizou o programa de exercícios.		Diferença estatisticamente significativa na atividade EMG para os músculos M e T em diversas situações

**Legenda: **F = Feminino; M = Masculino; TMO = Terapia Miofuncional Orofacial; GT = Grupo Tratado; UTG = Grupo Não Tratado; P0 = antes da cirurgia; P1 = 3 meses após a cirurgia; P2 = 6 meses após a cirurgia; P1** = antes da cirurgia; P2** = 6 meses após a cirurgia; P1*** = antes da cirurgia; P3*** = pós-operatório (3 anos a 3 anos e 8 meses); OHIP-14 = Perfil de Impacto na Saúde Bucal; M = Músculo masseter; T = Músculo Temporal

* Informações fornecidas pela autora do estudo incluído^(18)^

### Análise dos dados

A análise dos dados foi conduzida com o intuito de mapear os programas de intervenção miofuncional orofacial e cervical desenvolvidos para adultos com deformidades dentofaciais submetidos à cirurgia ortognática e verificar sua influência no sistema estomatognático.

A atuação fonoaudiológica no pós-operatório de cirurgia ortognática apresenta como objetivo a redução do edema facial, a promoção da sensibilidade orofacial, da mímica facial e da amplitude dos movimentos mandibulares, bem como a reintrodução gradual das consistências alimentares e a reabilitação das funções orofaciais^(7)^. Diante disso, na prática clínica é utilizado a TMO, composta por exercícios isométricos (contração estática), isotônicos (contração dinâmica) e de contrarresistência.

O planejamento terapêutico é constituído pela repetição e prática sistemática desses exercícios, com uma abordagem gradual em termos de frequência, volume (número de séries, repetições e sessões) e intervalo de descanso. Portanto, a análise das variáveis extraídas, visou examinar três principais desfechos: tipo de exercício (isométricos, isotônicos ou de contrarresistência), efeitos funcionais (funções orofaciais, qualidade de vida e saúde oral) e efeitos musculares (tônus e mobilidade).

A partir disso, após a extração dos dados, a análise dos resultados foi realizada em duas etapas: categorização dos estudos e análise dos desfechos observados. Dessa forma, constituiu-se um processo qualitativo e descritivo, dado o caráter heterogêneo dos estudos incluídos.

A análise referente aos resultados apresentados no processo de avaliação, seja com uso de métodos instrumentais, protocolos de avaliação miofuncional orofacial ou questionários de autoavaliação foram apresentados graficamente mediante um gráfico de bolhas, adequado para visualização de variáveis categóricas, ilustrado na Figura 1. Para sua construção, foi utilizada a linguagem Python, com emprego das bibliotecas Matplotlib e NumPy para manipulação de dados e visualização gráfica. O resultado da avaliação foi considerado positivo (com melhora dos padrões estruturais e funcionais do SE durante o período pós-cirurgia ortognática, representado por bolhas verdes) ou negativo (com piora dos padrões estruturais e funcionais do SE durante o período pós-cirurgia ortognática, representado por bolhas vermelhas). Cada estudo foi representado por uma bolha, posicionada nos eixos x e Y para indicar, respectivamente, a estratégia aplicada e o autor do estudo. A cor das bolhas foi utilizada para diferenciar os resultados e seu tamanho indica a amostra de indivíduos que realizaram Terapia Miofuncional Orofacial. A partir disso, o gráfico foi utilizado com o intuito de representar visualmente os resultados dos estudos incluídos nesta revisão, de forma clara e comparativa.

**Figura 1:** Gráfico de bolhas para variáveis categóricas para o resultado da Terapia Miofuncional Orofacial em adultos submetidos a cirurgia ortognática

Razão da exclusão:

Razão 1. Estudos nos quais a intervenção miofuncional orofacial e cervical é conduzida por profissionais de saúde sem formação em Fonoaudiologia

Razão 2. Estudos com adultos com histórico de alterações psiquiátricas, neurológicas, neuromusculares, neurodegenerativas ou oncológicas na região orofacial e/ou cervical

Razão 3. Estudos envolvendo adultos com fissura labiopalatina, disfagia orofaríngea, síndrome da apneia obstrutiva do sono, disfunção temporomandibular e/ou trauma facial

Razão 4. Estudos secundários, como resumos, revisões sistemáticas, revisões narrativas, revisões integrativas, meta-análises, cartas ao editor, diretrizes e guias de prática clínica

Razão 5. Estudos com amostras repetidas

Fonte: Page et al.^(25)^. Para mais informações, visite o site PRISMA^(26)^

Por fim, elaborou-se uma síntese narrativa quanto à descrição dos programas de intervenção miofuncional orofacial e cervical incluídos neste estudo, com intuito de apresentar a organização, composição e objetivos terapêuticos frente ao pós-operatório.

## RESULTADOS

### Seleção de fontes de evidências

Um total de 622 artigos nas bases de dados e na literatura cinzenta foram recuperados. Após a remoção das duplicatas, 559 citações foram identificadas. A partir da análise do título e do resumo, 543 artigos foram excluídos, resultando na identificação de 16 artigos, que foram objeto de recuperação e avaliação quanto à sua elegibilidade, sendo um dos estudos não recuperado na íntegra. Dentre estes, 10 foram excluídos, sendo que as justificativas para a exclusão e suas respectivas referências estão no Apêndice B. Portanto, cinco^(5,11,18-20)^ estudos foram considerados elegíveis para integrar esta revisão de escopo, conforme ilustrado na Figura 2.

**Figura 2:** Diagrama de fluxo da seleção dos estudos/fonte de evidências

### Características das fontes de evidências

Os cinco estudos^(5,11,18-20)^ elegíveis foram publicados em inglês, entre 2006 e 2018, e foram desenvolvidos no Brasil. A idade dos participantes variou entre 19 e 31 anos, com uma proporção média de 60% de participantes do sexo feminino e 40% do sexo masculino. Para composição das amostras, os estudos utilizaram a divisão entre grupos de intervenção e controle, empregando delineamento clínico randomizado^(5,11)^ ou de coorte^(18-20)^. O tamanho amostral variou entre 10 e 15 participantes, com períodos de seguimento de 15 dias a 6 meses.

### Resultados das fontes individuais de evidências

A Tabela 1 descreve os autores, país, ano de publicação, delineamentos metodológicos empregados, instrumentos ou tecnologias utilizadas para avaliação, além das características das intervenções, seja por meio de programas de intervenção ou exercícios miofuncionais, presentes nos cinco estudos elegíveis, bem como sintetiza desfechos observados após a intervenção.

As técnicas de avaliação incluíram Eletromiografia (EMG)^(11,20)^ e Ultrassonografia^(18,20)^, além de protocolos validados como Protocolo de Avaliação Miofuncional Orofacial (MBGR)^(5)^ e o Protocolo de Avaliação Miofuncional Orofacial com Escores Expandidos (AMIOFE-E)^(11)^. O questionário autorreferido empregado foi o *Oral Health Impact Profile* (OHIP-14)^(5)^.

De forma complementar aos protocolos validados ocorreu a utilização de avaliação com os seguintes objetos de investigação: o modo predominante na função respiratória (oral, nasal ou misto), a fase oral da deglutição, a mastigação, a fala, e aspectos de tonicidade e postura de lábios e língua^(18)^.

A avaliação foi conduzida após a realização da cirurgia ortognática, pré e pós intervenção miofuncional orofacial e cervical. Todavia, observa-se uma diversidade em seu intervalo, com variação de três meses^(5,11)^, seis meses^(11)^ e entre três anos e três anos e oito meses^(19,20)^ após a intervenção. Além disso, um estudo conduziu a avaliação no período pré-operatório, com uma média de três meses anteriores à intervenção cirúrgica, seguida de análise realizada entre seis a oito meses após a cirurgia ortognática^(18)^. Em relação ao número de sessões ou frequência, observamos uma diversidade nos resultados encontrados, com variação desde uma frequência semanal ao longo de oito a 15 semanas^(5,11)^ até um período estendido de seis meses^(18)^ ou 11 meses^(19,20)^.

No que concerne ao processo de intervenção, três estudos^(18-20)^ abordaram diretrizes pós-cirúrgicas, a prática de exercícios isométricos, isotônicos, posturais e funcionais, bem como o uso de estimulação sensorial tátil e térmica. Os dois estudos^(5,11)^ restantes incorporaram os exercícios isométricos e isotônicos à estimulação tátil-cinestésica e térmica, com o intuito de evidenciar as suas contribuições para a melhora funcional do sistema estomatognático.

Com o intuito de verificar o efeito da TMO nas funções orofaciais e na qualidade de vida de indivíduos submetidos à cirurgia ortognática^(5)^ foi empregada intervenção fonoaudiológica mediante aplicação de: estimulação tátil-cinestésica e térmica no terço facial inferior; exercícios isotônicos em lábios, língua e mandíbula; exercícios para adequação de tônus de língua, lábios, bochechas e mento e exercícios para otimizar os aspectos morfológicos dos lábios, tendo em vista a frequência de lábio superior encurtado ou lábio evertido.

A abordagem do treinamento funcional^(5)^ foi concebida com o intuito de melhorar a postura habitual de lábios, mandíbula e língua, além de otimizar as funções orofaciais, como: respiração mediante estimulação da respiração nasal e treinamento do trato respiratório médio inferior; mastigação por meio da implementação do padrão mastigatório bilateral alternado ou simultâneo; deglutição por interferência da adequação de tônus e mobilidade dos lábios e língua; e fala (origem fonética) por intermédio da adequação da mobilidade, tônus e propriocepção da língua e do padrão articulatório. Ademais, com o objetivo de manutenção do equilíbrio orofacial e estético funcional, houve o emprego de exercícios que auxiliassem na expressividade durante a comunicação oral.

O *Post Orthognathic surgery therapy Protocol* ^(11)^, desenvolvido para analisar os aspectos clínicos e eletromiográficos da função mastigatória três e seis meses após a cirurgia ortognática, refere-se a um plano estruturado. Em sua composição há os seguintes elementos: nas duas primeiras sessões, foram conduzidas atividades de estimulação tátil e térmica, associadas à propriocepção da deglutição e a exercícios isotônicos, mantendo-se, estas últimas abordagens, até a oitava e terceira sessões, respectivamente. Na segunda sessão, foram introduzidos exercícios destinados ao desenvolvimento do tônus muscular, à correção da postura habitual da língua e ao treinamento do padrão mastigatório bilateral simultâneo, com duração mantida até a oitava, quarta e terceira sessões, respectivamente. Na quarta sessão, o treinamento do padrão mastigatório bilateral alternado e a abordagem fonética da fala foram incorporados, com manutenção até a oitava sessão. A nona sessão foi designada para a reavaliação do progresso terapêutico, enquanto na décima sessão, as orientações finais foram fornecidas aos participantes.

Com o propósito de determinar a influência do tratamento interdisciplinar em pacientes classe III e as alterações bilaterais na espessura do músculo masseter em situações de repouso e mordida^(18)^, bem como analisar o impacto do tratamento interdisciplinar envolvendo ortodontia, cirurgia e terapia miofuncional orofacial em pacientes com deformidade dentofacial Classe III na atividade eletromiográfica dos músculos mastigatórios^(20)^ e na espessura do músculo masseter^(19)^ após três anos da correção cirúrgica, ocorreu o desenvolvimento de um plano estruturado.

Diante disso, a TMO pós-cirúrgica^(18-20)^ foi constituída pelas seguintes intervenções: aplicação de compressas mornas e massagem a partir de duas semanas após a cirurgia, visando a redução do edema facial, o relaxamento dos músculos levantadores da mandíbula e a melhoria dos movimentos mandibulares. Orientações relativas à consistência alimentar foram fornecidas, iniciando com uma dieta líquida e progredindo gradualmente para uma dieta sólida. Para os pacientes que relataram dor ou desconforto na articulação temporomandibular (ATM), recomendações foram feitas para manter uma dieta leve e praticar a mastigação bilateral simultânea. Além disso, foram prescritos exercícios específicos para promover a recuperação gradual dos movimentos mandibulares, incluindo técnicas para fortalecer e melhorar a postura dos lábios e da língua. Quando identificadas alterações na deglutição, mastigação ou fala, foram realizados exercícios corretivos direcionados a esses aspectos específicos, visando uma reabilitação abrangente e personalizada para cada paciente^(18-20)^. Ademais, como parte do processo de reabilitação, ocorreu a aplicação de estimulação sensorial tátil e térmica^(18-20)^.

É essencial salientar que, ao longo das sessões de intervenção, a função mastigatória foi praticada com alimentos naturais, com uma progressão gradual da consistência alimentar durante o período pós-operatório. O treinamento funcional direcionou-se ao alongamento dos músculos elevadores da mandíbula e do músculo masseter, com o objetivo de restaurar os movimentos de abertura, lateralidade e protrusão mandibular, frequentemente comprometidos pela cirurgia ortognática mandibular^(19)^.

### Síntese dos resultados

Os resultados do processo de avaliação foram apresentados no gráfico de bolhas (Figura 1), que permitiram uma visualização clara e comparativa dos dados. Observe-se que a maioria dos estudos apresentaram resultados positivos, indicados pelas bolhas verdes, que representam melhorias nos padrões estruturais e funcionalidade do SE durante o período pós-cirurgia ortognática. Já os estudos com resultados negativos, indicados por bolhas vermelhas, mostraram piora nos padrões. O tamanho das bolhas refletiu a amostra de cada estudo, permitindo uma análise comparativa da efetividade da Terapia Miofuncional Orofacial.

Diante desse cenário, regimes de exercícios específicos foram elaborados com o objetivo de induzir melhorias nos padrões funcionais do SE durante o período pós-cirurgia ortognática. No âmbito deste estudo, foram identificados resultados positivos que corroboram com a eficácia das intervenções propostas, destaca-se: o incremento substancial na espessura dos músculos temporal direito e esquerdo^(20)^ e masseter direito e esquerdo^(18,19)^ no repouso e durante a mordida^(18)^, melhoria significativa no desempenho das funções orofaciais^(5)^, tais como respiração, mastigação, deglutição e fala, sugerindo uma otimização da funcionalidade do sistema estomatognático; aumento no escore do Protocolo OHIP-14^(5)^ e uma pontuação máxima na Avaliação de Saúde Oral (OMS-E)^(11)^, indicando uma melhor percepção da saúde oral por parte dos participantes após a intervenção; e melhorias no tônus do lábio inferior, mobilidade da língua e aumento no número de ciclos mastigatórios^(11)^.

Como resultados neutros, destacam-se a ausência de alterações nos movimentos da cabeça e no escape alimentar^(11)^, de diferenças entre os lados na espessura do músculo masseter^(18)^, e de diferença estatística para o masseter esquerdo em contração ao realizar mordida^(19)^. Embora tenha sido observado um aumento numérico de indivíduos que alcançaram a adequação na mobilidade labial, não foram identificadas diferenças estatisticamente significativas^(11)^.

Como resultados negativos, ressalta-se a ausência de progresso nos parâmetros de mastigação, deglutição e fala no grupo que não foi submetido a TMO^(5)^; ausência de diferença para a mordida e a presença de indivíduos que manifestaram postura de cabeça alterada no grupo submetido a TMO^(11)^; inexistência de significância estatística para a mordida, elevado número de participantes com alterações no tônus do lábio inferior e na mobilidade de lábios e língua no grupo que não foi submetido a TMO^(11)^.

## DISCUSSÃO

Esta revisão de escopo apresentou como objetivo mapear as evidências sobre os programas de intervenção miofuncional orofacial e cervical em adultos com deformidades dentofaciais submetidos à cirurgia ortognática. Diante disso, a importância da temática reside na necessidade de compreender os resultados funcionais e estruturais da terapia miofuncional orofacial na reabilitação de pacientes submetidos à cirurgia ortognática.

A caracterização do perfil amostral em relação à idade e sexo predominante pode ser justificada pelo processo de desenvolvimento e crescimento craniofacial. A definição das características esqueléticas craniofaciais ocorre durante a puberdade. Todavia, é na fase adulta que as deformidades dentofaciais, consolidadas pelo término do crescimento craniofacial^(21)^, determinarão padrões funcionais adaptativos quanto à postura oral habitual, à respiração, à mastigação, deglutição e pontos articulatórios de fala, compatíveis com a característica anatômica apresentada. A variável sexo emerge como um fator interveniente no desenvolvimento esquelético, dado que o sexo feminino tende a atingir sua forma facial adulta de maneira precoce em comparação ao sexo masculino^(22)^. Além disso, a presença de deformidade dentofacial pode resultar em uma maior propensão à dor orofacial no gênero feminino, acompanhada de uma preocupação acentuada com os aspectos estéticos e sociais, quando comparado com o sexo masculino^(23)^.

Os estudos de intervenção são investigações primárias que envolvem a manipulação controlada e intencional de um fator de exposição, visando analisar os efeitos das alterações promovidas. Esses estudos têm um delineamento prospectivo e são frequentemente utilizados para conduzir ensaios clínicos randomizados e controlados^(24)^. Nesta revisão, foram selecionados estudos de caráter terapêutico para assegurar a comparabilidade entre múltiplas variáveis e reduzir o risco de vieses de seleção e confundimento.

A partir disso, a revisão concentrou-se em mapear e descrever os resultados relatados dos programas de intervenção miofuncional orofacial e cervical, bem como nos exercícios miofuncionais, sobre as funções e estruturas orofaciais em pacientes submetidos à cirurgia ortognática. Além dos ensaios clínicos, foram incluídos três estudos com delineamento coorte^(18-20)^, delineamento que contribui com a compreensão dos efeitos de longo prazo e na identificação de associações causais em contextos em que ensaios clínicos podem não ser viáveis ou éticos. Contudo, uma limitação associada aos estudos de coorte é sua reduzida capacidade de estabelecer causalidade direta, em comparação com ensaios clínicos randomizados, devido à possibilidade de fatores de confusão residuais^(24)^.

Nos estudos elegíveis, a avaliação das estruturas e funções orofaciais foi conduzida por meio de métodos instrumentais, como a Eletromiografia de Superfície (EMGs)^(11,20)^ e a Ultrassonografia^(18,19)^. Esses métodos fornecem uma abordagem quantitativa e complementar essencial para o diagnóstico clínico dos distúrbios miofuncionais orofaciais. A ultrassonografia é empregada como uma técnica morfométrica para medir a espessura dos músculos orofaciais e monitorar os movimentos da língua durante a mastigação, deglutição e fala. Em contrapartida, a EMGs é um instrumento eletrofisiológico utilizado para captar a atividade elétrica dos músculos orofaciais e cervicais durante o repouso, em contração isométrica máxima e em tarefas específicas das funções orofaciais.

Além das avaliações instrumentais, observa-se a utilização da avaliação miofuncional orofacial mediante o emprego dos instrumentos validados, intitulados por: Protocolo de Avaliação Miofuncional Orofacial (MBGR)^(5)^ e Protocolo de Avaliação Miofuncional Orofacial com Escores Expandidos (AMIOFE-E)^(11)^. Para uma avaliação miofuncional orofacial detalhada e abrangente das condições anatômicas e funcionais do sistema estomatognático, faz-se imprescindível a aplicação desses protocolos de avaliação.

Embora as avaliações instrumentais ofereçam uma compreensão clara da anatomofisiologia dos sistemas envolvidos e forneçam biofeedback sobre a atividade muscular, é importante reconhecer que o alto custo dessas tecnologias pode limitar sua acessibilidade e uso generalizado. Além disso, o planejamento terapêutico eficaz é constituído por uma avaliação clínica e avaliação miofuncional orofacial minuciosa e abrangente. Dessa forma, a combinação de avaliações clínicas e instrumentais, permite uma abordagem terapêutica mais precisa e personalizada, o que pode otimizar os resultados terapêuticos e a qualidade de vida dos pacientes.

A intervenção miofuncional orofacial e cervical desenvolvida para indivíduos com deformidades dentofaciais submetidos a cirurgia ortognática é constituída por diretrizes pós-cirúrgicas, estimulação sensorial tátil e térmica e a prática de exercícios isométricos, isotônicos, posturais e funcionais^(18-20)^, bem como por exercícios isométricos e isotônicos associados à estimulação tátil-cinestésica e térmica^(5,11)^. Diante disso, o presente estudo visa mapear a síntese de evidências sobre programas de intervenção miofuncional orofacial e cervical desenvolvidos para adultos submetidos à cirurgia ortognática e sua influência no sistema estomatognático.

Na equipe interdisciplinar de cirurgia ortognática, o papel do fonoaudiólogo é fundamental, especialmente na avaliação do sistema miofuncional orofacial no pré-operatório e pós-operatório^(21)^. A avaliação miofuncional orofacial pré-operatória, realizada pelos cinco estudos elegíveis desta revisão^(5,11,18-20)^, apresenta o objetivo de identificar e caracterizar as alterações musculares e funcionais presentes, o que permite o desenvolvimento de protocolos específicos de intervenção, com orientações e exercícios direcionados aos distúrbios miofuncionais orofaciais identificados, além de preparar o paciente para a cirurgia e auxiliar na recuperação pós-cirúrgica. Ademais, a avaliação pré-cirúrgica fornece, à equipe e ao paciente, o fator comparativo entre as avaliações e períodos analisados, com intuito de verificar as características, compensações e adaptações do sistema estomatognático decorrentes da DDF^(1,21,22)^.

Com a compressão das condições anatômicas e funcionais do sistema estomatognático, haverá a manutenção da intervenção fonoaudiológica no pré e pós-operatório. Na fase pré-operatória, as intervenções são recomendadas 30 a 60 dias antes da cirurgia, com o intuito de fornecer orientações e esclarecimentos quanto à percepção dos mecanismos e padrões musculares adequados, em repouso e durante a execução das funções orofaciais^(21,22)^. Além de auxiliar em um novo sistema proprioceptivo e otimizar as condições funcionais do sistema estomatognático em preparação para as modificações anatômicas subsequentes à cirurgia.

O período pós-cirúrgico, inicia-se nas primeiras 24 horas após a cirurgia ou com aproximadamente 20 a 60 dias após o procedimento^(7,21,22)^. Essa fase é caracterizada por uma abordagem multifacetada e inclui estratégias para redução do edema facial, reabilitação da sensibilidade orofacial, estimulação da mímica facial e aumento da amplitude dos movimentos mandibulares^(7,21)^. Analogamente, um protocolo gradual de reintrodução das consistências alimentares é estabelecido, bem como há o ajuste e a adequação das funções orofaciais, com o intuito de alcançar o restabelecimento eficaz do sistema estomatognático. Sob essa perspectiva, o início do processo de intervenção dos estudos incluídos nesta revisão, está alinhado ao período preconizado pela literatura. Os programas de intervenção descritos nos estudos elegíveis, são compostos por exercícios isométricos, isotônicos, posturais e funcionais, bem como estímulos tátil-cinestésicos e térmicos. Essas técnicas são empregadas para potencializar a eficácia do tratamento e facilitar uma recuperação abrangente.

Um estudo incluído^(19)^ nesta revisão avaliou o impacto da intervenção miofuncional sobre a espessura do músculo masseter esquerdo em contração em 28 participantes, sendo 13 submetidos a um protocolo interdisciplinar (ortodontia, cirurgia e terapia miofuncional orofacial) para deformidade dentofacial Classe III. Após três anos, os resultados mostraram que a espessura do masseter esquerdo em contração nos pacientes tratados não diferiu significativamente da observada em indivíduos sem alterações esqueléticas, indicando uma aproximação ao padrão de normalidade. Embora não tenha sido observado um aumento significativo na espessura muscular, o tratamento promoveu uma harmonização da espessura em repouso entre os lados e estabeleceu condições favoráveis à oclusão dentária e à flexibilidade muscular. Os achados reforçam a importância da terapia integrada para a reabilitação funcional e estética em pacientes com deformidade Classe III.

Dessa forma, a combinação de estratégias terapêuticas e a adesão a protocolos de intervenção são cruciais para a otimização dos resultados pós-cirúrgicos. A integração dessas práticas destaca a necessidade de um manejo sistemático, personalizado e individual, durante o período pós-operatório, refletindo um modelo de cuidados baseado em evidências para a reabilitação orofacial e garantindo a máxima recuperação funcional e estética do paciente.

### Limitações

A extensa busca na literatura de artigos sobre o tema proposto que englobou bases de dados internacionais e multidisciplinares, seguiu rigorosamente as diretrizes da revisão de escopo. A síntese dos dados e a consulta da literatura cinzenta, são pontos fortes evidentes do presente estudo, juntamente com a inclusão de estudos que envolvem as funções SE, atuação da fonoaudiologia e intervenções na cirurgia ortognática.

Entre as limitações, a mais evidente é a lacuna substancial dos achados juntamente com a variabilidade dos programas de intervenção e a ausência de protocolos validados para a avaliação clínica miofuncional orofacial e para a avaliação instrumental das estruturas e funções orofaciais em pacientes submetidos a cirurgia ortognática. Essa lacuna limita a generalização dos resultados e a aplicação prática das descobertas.

Adicionalmente, observa-se uma deficiência no detalhamento dos exercícios utilizados nas intervenções, com foco predominantemente nos objetivos gerais e sem especificação adequada sobre a quantidade necessária de sessões e repetições para garantir uma eficácia terapêutica efetiva.

É importante destacar que os estudos incluídos apresentam heterogeneidade metodológica, como diferenças nos desenhos de estudo, tamanhos amostrais reduzidos e variações nos critérios de avaliação das intervenções miofuncionais. Essa diversidade dificulta a comparação direta dos resultados e a consolidação de conclusões robustas. Ademais, a maioria dos estudos carece de acompanhamentos de longo prazo, o que limita a compreensão dos efeitos sustentados das intervenções propostas.

Por fim, é importante ressaltar que revisões de escopo, diferentemente das revisões sistemáticas, não exigem uma avaliação crítica da qualidade metodológica dos estudos incluídos. Portanto, as limitações inerentes à interpretação dos resultados devem ser reconhecidas e consideradas.

### Sugestões para pesquisas futuras

Com base na literatura revisada, é possível compreender por que os estudos primários têm definido objetivos e regimes de exercícios específicos para melhorar o SE de pacientes com deformidades dentofaciais submetidos a cirurgia ortognática. No entanto, ainda há uma necessidade de análise mais específica, detalhada e integrada. Dessa forma, caso os estudos primários continuem a ser desenvolvidos da mesma forma, uma resposta definitiva permanece indefinida. Portanto, com base em nossos achados, propomos as seguintes direções para pesquisas futuras e considerações para a prática clínica.

Nessa perspectiva, para abordar essas lacunas e aperfeiçoar a prática clínica, é imperativo validar um instrumento de avaliação miofuncional orofacial para uso pré e pós-operatório em pacientes submetidos à cirurgia ortognática. Este instrumento deve ser desenvolvido para estabelecer padrões claros de avaliação, caracterizar de forma detalhada os distúrbios miofuncionais orofaciais e possibilitar a formulação de planos terapêuticos individualizados e fundamentados em evidências.

Além disso, recomenda-se o desenvolvimento de um programa de intervenção miofuncional orofacial e cervical para adultos submetidos à cirurgia ortognática. Este programa deve definir a atuação do fonoaudiólogo na equipe interdisciplinar, os períodos de intervenção, estabelecer objetivos terapêuticos específicos e identificar os parâmetros a serem avaliados. A implementação de um programa estruturado e baseado em evidências proporcionará diretrizes práticas robustas, orientando a prática clínica quanto à duração da intervenção, parâmetros avaliados e metas a serem atingidas.

Estas estratégias visam aprimorar a eficácia das intervenções miofuncionais, promovendo um planejamento terapêutico mais preciso e baseado em evidências, e contribuindo assim para uma recuperação mais eficaz do sistema estomatognático no pós-cirúrgico, com redução de recidivas.

## CONCLUSÃO

As sínteses de evidências identificadas no mapeamento indicam que os programas de intervenção miofuncional orofacial e cervical, desenvolvidos para adultos com DDF submetidos a cirurgia ortognática, auxiliam na recuperação e na melhoria da funcionalidade do sistema estomatognático.

Os estudos elegíveis, que incluem tanto delineamentos clínicos randomizados quanto estudos de coorte, demonstram que intervenções estruturadas, compostas por exercícios isométricos, isotônicos, posturais e funcionais, resultam em melhorias substanciais na espessura muscular do masseter, no desempenho das funções orofaciais e na percepção geral da saúde oral.

Dessa forma, as intervenções miofuncionais orofaciais são fundamentais na reabilitação pós-cirúrgica, visto que contribuem para a otimização da funcionalidade do sistema estomatognático, ainda que a variabilidade nos resultados indique a necessidade de abordagens individualizadas e com avaliações fundamentadas e validadas.

## Apêndice A Estratégias de busca nas bases de dados e literatura cinzenta

**Table:**

**Base de dados**	**Busca** (25 de outubro de 2023)
**Embase**	#1 'myofunctional therapy'/exp OR 'myofunctional therapy' OR 'myofunctional therapies' OR 'orofacial myotherapy' OR 'oral myotherapy' OR 'orofacial myology' OR 'oral exercise' OR 'promotion program' OR 'long-term care prevention programs' OR 'resistance training'/exp OR 'resistance training' OR 'strength training'/exp OR 'strength training' OR 'exercise program'/exp OR 'exercise program' OR 'exercise therapy'/exp OR 'exercise therapy' OR 'remedial exercise' OR 'remedial exercises' OR 'exercise therapies' OR 'rehabilitation exercise' OR 'rehabilitation'/exp OR 'rehabilitation' OR 'habilitation' OR 'speech therapy'/exp OR 'speech therapy' OR 'speech therapies' #2 'dentofacial deformities' OR 'dentofacial deformity' OR 'dentofacial abnormalities' OR 'dentofacial abnormality' OR 'orthognathic surgery' OR 'orthognathic surgeries' OR 'orthognathic surgical procedures' OR 'orthognathic surgical procedure' OR 'maxillo-mandibular surgery' OR 'maxillo mandibular surgery' OR 'maxillofacial orthognathic surgery' OR 'maxillofacial orthognathic surgeries' OR 'surgical-orthodontic' OR 'orthognathic' OR 'orthodontic-surgical' OR 'orthodontic-surgery' OR 'jaw surgeries' OR 'jaw surgery' #3 'stomatognathic system' OR 'stomatognathic systems' OR 'masticatory system' OR 'masticatory systems' OR 'mastication' OR 'chewing' OR 'sucking behavior' OR 'sucking behaviors' OR 'sucking' OR 'deglutition' OR 'deglutitions' OR 'swallowing' OR 'swallowings' OR 'speech' OR 'public speaking' OR 'respiration' OR 'breathing' #4 #1 AND #2 AND #3 #5 #4 AND [embase]/lim NOT ([embase]/lim AND [medline]/lim)
**LILACS**	(“myofunctional therapy” OR “myofunctional therapies” OR “orofacial myotherapy” OR “oral myotherapy” OR “orofacial myology” OR “oral exercise” OR “promotion program” OR “long-term care prevention programs” OR “resistance training” OR “strength training” OR “exercise program” OR “exercise therapy” OR “remedial exercise” OR “remedial exercises” OR “exercise therapies” OR “rehabilitation exercise” OR “rehabilitation” OR “habilitation” OR “speech therapy” OR “speech therapies” OR “terapia miofuncional” OR “miologia orofacial” OR “treinamento de força” OR “treino de força” OR “treino por exercício” OR “exercício de reabilitação” OR “exercício terapêutico” OR “reabilitação” OR “habilitação” OR “reabilitação da fala” OR “reeducação da fala” OR “treinamento de fala” OR “terapia miofuncional” OR “entrenamiento de fuerza” OR “terapia por ejercicio” OR “rehabilitación” OR “logopedia”) AND (“Dentofacial Deformities” OR “Dentofacial Deformity” OR “Dentofacial Abnormalities” OR “Dentofacial Abnormality” OR “Orthognathic Surgery” OR “Orthognathic Surgery” OR “Orthognathic Surgeries” OR “Maxillofacial Orthognathic Surgery” OR “Orthognathic Surgical Procedures” OR “Orthognathic Surgical Procedure” OR “Maxillo-Mandibular Surgery” OR “Maxillo Mandibular Surgery” OR “Maxillofacial Orthognathic Surgery” OR “Maxillofacial Orthognathic Surgeries” OR “surgical-orthodontic” OR “Orthognatic” OR “orthodontic-surgical” OR “orthodontic-surgery” OR “jaw surgeries” OR “jaw surgery” OR “deformidades dentofaciais” OR “cirurgia ortognótica” OR “procedimentos cirúrgicos ortognáticos” OR “cirurgia de mandíbula” OR “cirurgia mandibular” OR “cirurgia maxilomandibular” OR “cirurgia maxilomandibulares” OR “cirurgias ortognática maxilofaciais” OR “deformidades dentofaciales” OR “procedimentos quirúrgicos ortognáticos”) AND (“Stomatognathic System” OR “Stomatognathic Systems” OR “Masticatory System” OR “Masticatory Systems” OR “Mastication” OR “Chewing” OR “Sucking Behavior” OR “Sucking Behavior” OR “sucking” OR “Deglutition” OR “Deglutitions” OR “Swallowing” OR “Swallowings” OR “Speech” OR “Public Speaking” OR “Respiration” OR “Breathing” OR “sistema estomatognático” OR “sistema mastigatório” OR “mastigação” OR “Comportamento de Sucção” OR “Conducta en la Lactancia” OR “deglutição” OR “fala” OR “respiração” OR “sistema estomatognático” OR “sistema masticatorio” OR “masticación” OR “conducta en la lactancia” OR “deglución” OR “habla” OR “respiración”)
**PubMed/ Medline**	(“myofunctional therapy”[Mesh] OR “myofunctional therapy” OR “myofunctional therapies” OR “orofacial myotherapy” OR “oral myotherapy” OR “orofacial myology” OR “oral exercise” OR “promotion program” OR “long-term care prevention programs” OR “resistance training”[Mesh] OR “strength training” OR “exercise program” OR “exercise therapy”[Mesh] OR “remedial exercise” OR “rehabilitation exercise” OR “rehabilitation”[Mesh] OR “habilitation” OR “speech therapy” OR “speech therapies”) AND (“Dentofacial Deformities”[Mesh] OR “Dentofacial Deformities” OR “Dentofacial Deformity” OR “Dentofacial Abnormalities” OR “Dentofacial Abnormality” OR “Orthognathic Surgery”[Mesh] OR “Orthognathic Surgery” OR “Orthognathic Surgeries” OR “Maxillofacial Orthognathic Surgery” OR “Orthognathic Surgical Procedures” OR “Orthognathic Surgical Procedures”[Mesh] OR “Orthognathic Surgical Procedures” OR “Orthognathic Surgical Procedure” OR “Maxillo-Mandibular Surgery” OR “Maxillo Mandibular Surgery” OR “Maxillofacial Orthognathic Surgery” OR “Maxillofacial Orthognathic Surgeries” OR “surgical-orthodontic” OR “Orthognatic” OR “orthodontic-surgical” OR “orthodontic-surgery”) AND (“Stomatognathic System”[MeSH] OR “Stomatognathic System” OR “Stomatognathic Systems” OR “Masticatory System” OR “Masticatory Systems” OR “Mastication”[MesH] OR “Mastication” OR “Chewing” OR “Sucking Behavior”[Mesh] OR “Sucking Behavior” OR “Sucking Behaviors” OR “sucking” OR “Deglutition”[Mesh] OR “Deglutition” OR “Deglutitions” OR “Swallowing” OR “Swallowings” OR “Speech”[Mesh] OR “Speech” OR “Public Speaking” OR “Respiration”[Mesh] OR “Breathing”)
**Scopus**	(TITLE-ABS-KEY (“myofunctional therapy” OR “myofunctional therapies” OR “orofacial myotherapy” OR “oral myotherapy” OR “orofacial myology” OR “oral exercise” OR “promotion program” OR “long-term care prevention programs” OR “resistance training” OR “strength training” OR “exercise program” OR “exercise therapy” OR “remedial exercise” OR “rehabilitation exercise” OR “rehabilitation” OR “habilitation” OR “speech therapy” OR “speech therapies”) AND TITLE-ABS-KEY (“Dentofacial Deformities” OR “Dentofacial Deformity” OR “Dentofacial Abnormalities” OR “Dentofacial Abnormality” OR “Orthognathic Surgery” OR “Orthognathic Surgeries” OR “Maxillofacial Orthognathic Surgery” OR “Orthognathic Surgical Procedures” OR “Orthognathic Surgical Procedure” OR “Maxillo-Mandibular Surgery” OR “Maxillo Mandibular Surgery” OR “Maxillofacial Orthognathic Surgery” OR “Maxillofacial Orthognathic Surgeries” OR “surgical-orthodontic” OR “Orthognatic” OR “orthodontic-surgical” OR “orthodontic-surgery”) AND TITLE-ABS-KEY (“Stomatognathic System” OR “Stomatognathic Systems” OR “Masticatory System” OR “Masticatory Systems” OR “Mastication” OR “Chewing” OR “Sucking Behavior” OR “Sucking Behavior’” OR “sucking” OR “Deglutition” OR “Deglutitions” OR “Swallowing” OR “Swallowings” OR “Speech” OR “Public Speaking” OR “Respiration” OR “Breathing”)
**Web of Science**	(“myofunctional therapy” OR “myofunctional therapies” OR “orofacial myotherapy” OR “oral myotherapy” OR “orofacial myology” OR “oral exercise” OR “promotion program” OR “long-term care prevention programs” OR “resistance training” OR “strength training” OR “exercise program” OR “exercise therapy” OR “remedial exercise” OR “rehabilitation exercise” OR “rehabilitation” OR “habilitation” OR “speech therapy” OR “speech therapies”) (Tópico) AND (“Dentofacial Deformities” OR “Dentofacial Deformity” OR “Dentofacial Abnormalities” OR “Dentofacial Abnormality” OR “Orthognathic Surgery” OR “Orthognathic Surgeries” OR “Maxillofacial Orthognathic Surgery” OR “Orthognathic Surgical Procedures” OR “Orthognathic Surgical Procedure” OR “Maxillo-Mandibular Surgery” OR “Maxillo Mandibular Surgery” OR “Maxillofacial Orthognathic Surgery” OR “Maxillofacial Orthognathic Surgeries” OR “surgical-orthodontic” OR “orthognathic” OR “orthodontic-surgical” OR “orthodontic-surgery”) (Tópico) AND (“Stomatognathic System” OR “Stomatognathic Systems” OR “Masticatory System” OR “Masticatory Systems” OR “Mastication” OR “Chewing” OR “Sucking Behavior” OR “Sucking Behavior” OR “sucking” OR “Deglutition” OR “deglutition” OR “swallowing” OR “Swallowings” OR “Speech” OR “Public Speaking” OR “Respiration” OR “Breathing”)
**Cochrane**	(“myofunctional therapy” OR “myofunctional therapies” OR “orofacial myotherapy” OR “oral myotherapy” OR “orofacial myology” OR “oral exercise” OR “promotion program” OR “long-term care prevention programs” OR “resistance training” OR “strength training” OR “exercise program” OR “exercise therapy” OR “remedial exercise” OR “rehabilitation exercise” OR “rehabilitation” OR “habilitation” OR “speech therapy” OR “speech therapies”) in Title Abstract Keyword AND (“Dentofacial Deformities” OR “Dentofacial Deformity” OR “Dentofacial Abnormalities” OR “Dentofacial Abnormality” OR “Orthognathic Surgery” OR “Orthognathic Surgeries” OR “Maxillofacial Orthognathic Surgery” OR “Orthognathic Surgical Procedures” OR “Orthognathic Surgical Procedure” OR “Maxillo-Mandibular Surgery” OR “Maxillo Mandibular Surgery” OR “Maxillofacial Orthognathic Surgery” OR “Maxillofacial Orthognathic Surgeries” OR “surgical-orthodontic” OR “orthognathic” OR “orthodontic-surgical” OR “orthodontic-surgery”) in Title Abstract Keyword AND (“Stomatognathic System” OR “Stomatognathic Systems” OR “Masticatory System” OR “Masticatory Systems” OR “Mastication” OR “Chewing” OR “Sucking Behavior” OR “Sucking Behavior” OR “sucking” OR “Deglutition” OR “deglutition” OR “Swallowing” OR “swallowing” OR “Speech” OR “Public Speaking” OR “Respiration” OR “Breathing”)
**LIVIVO**	(“myofunctional therapy” OR “myofunctional therapies” OR “orofacial myotherapy” OR “oral myotherapy” OR “orofacial myology” OR “oral exercise” OR “promotion program” OR “long-term care prevention programs” OR “resistance training” OR “strength training” OR “exercise program” OR “exercise therapy” OR “remedial exercise” OR “rehabilitation exercise” OR “rehabilitation” OR “habilitation” OR “speech therapy” OR “speech therapies”) AND (“Dentofacial Deformities” OR “Dentofacial Deformity” OR “Dentofacial Abnormalities” OR “Dentofacial Abnormality” OR “Orthognathic Surgery” OR “Orthognathic Surgeries” OR “Maxillofacial Orthognathic Surgery” OR “Orthognathic Surgical Procedures” OR “Orthognathic Surgical Procedure” OR “Maxillo-Mandibular Surgery” OR “Maxillo Mandibular Surgery” OR “Maxillofacial Orthognathic Surgery” OR “Maxillofacial Orthognathic Surgeries” OR “surgical-orthodontic” OR “orthognathic” OR “orthodontic-surgical” OR “orthodontic-surgery”) AND (“Stomatognathic System” OR “Stomatognathic Systems” OR “Masticatory System” OR “Masticatory Systems” OR “Mastication” OR “Chewing” OR “Sucking Behavior” OR “Sucking Behavior” OR “sucking” OR “Deglutition” OR “deglutition” OR “swallowing” OR “Swallowings” OR “Speech” OR “Public Speaking” OR “Respiration” OR “Breathing”)
**Google Scholar**	(“myofunctional therapy” OR “exercise therapy”) AND (“dentofacial deformities” OR “orthognathic surgery”) filetype:PDF
**ProQuest**	NOFT (“myofunctional therapy” OR “myofunctional therapies” OR “orofacial myotherapy” OR “oral myotherapy” OR “orofacial myology” OR “oral exercise” OR “promotion program” OR “long-term care prevention programs” OR “resistance training” OR “strength training” OR “exercise program” OR “exercise therapy” OR “remedial exercise” OR “rehabilitation exercise” OR “rehabilitation” OR “habilitation” OR “speech therapy” OR “speech therapies”) AND NOFT (“Dentofacial Deformities” OR “Dentofacial Deformity” OR “Dentofacial Abnormalities” OR “Dentofacial Abnormality” OR “Orthognathic Surgery” OR “Orthognathic Surgeries” OR “Maxillofacial Orthognathic Surgery” OR “Orthognathic Surgical Procedures” OR “Orthognathic Surgical Procedure” OR “Maxillo-Mandibular Surgery” OR “Maxillo Mandibular Surgery” OR “Maxillofacial Orthognathic Surgery” OR “Maxillofacial Orthognathic Surgeries” OR “surgical-orthodontic” OR “orthognathic” OR “orthodontic-surgical” OR “orthodontic-surgery”) AND NOFT (“Stomatognathic System” OR “Stomatognathic Systems” OR “Masticatory System” OR “Masticatory Systems” OR “Mastication” OR “Chewing” OR “Sucking Behavior” OR “Sucking Behavior” OR “sucking” OR “Deglutition” OR “deglutition” OR “swallowing” OR “Swallowings” OR “Speech” OR “Public Speaking” OR “Respiration” OR “Breathing”)
**Biblioteca Digital Brasileira de Teses e Dissertações (BDTD)**	(“myofunctional therapy” OR “myofunctional therapies” OR “orofacial myotherapy” OR “oral myotherapy” OR “orofacial myology” OR “oral exercise” OR “promotion program” OR “long-term care prevention programs” OR “resistance training” OR “strength training” OR “exercise program” OR “exercise therapy” OR “remedial exercise” OR “rehabilitation exercise” OR “rehabilitation” OR “habilitation” OR “speech therapy” OR “speech therapies”) AND (“Dentofacial Deformities” OR “Dentofacial Deformity” OR “Dentofacial Abnormalities” OR “Dentofacial Abnormality” OR “Orthognathic Surgery” OR “Orthognathic Surgeries” OR “Maxillofacial Orthognathic Surgery” OR “Orthognathic Surgical Procedures” OR “Orthognathic Surgical Procedure” OR “Maxillo-Mandibular Surgery” OR “Maxillo Mandibular Surgery” OR “Maxillofacial Orthognathic Surgery” OR “Maxillofacial Orthognathic Surgeries” OR “surgical-orthodontic” OR “orthognathic” OR “orthodontic-surgical” OR “orthodontic-surgery”) AND (“Stomatognathic System” OR “Stomatognathic Systems” OR “Masticatory System” OR “Masticatory Systems” OR “Mastication” OR “Chewing” OR “Sucking Behavior” OR “Sucking Behavior” OR “sucking” OR “Deglutition” OR “deglutition” OR “swallowing” OR “Swallowings” OR “Speech” OR “Public Speaking” OR “Respiration” OR “Breathing”)

## Apêndice B Artigos excluídos e motivo da exclusão (n = 10)

**Table:**

**Autor**	**Motivos de exclusão**
Altmann^1^	4
Costa^2^	3
Gallerano et al.^3^	3
Lichnowska e Kozakiewicz^4^	4
Mangilli^5^	3
Min et al.^6^	1*
Ohba et al.^7^	1*
Trawitzki et al.^8^	5
Tyndor et al.^9^	3
Watanabe et al.^10^	1

**Legenda:** 1. Estudos nos quais a intervenção miofuncional orofacial e cervical é conduzida por profissionais de saúde sem formação em Fonoaudiologia; 2. Estudos com adultos com histórico de alterações psiquiátricas, neurológicas, neuromusculares, neurodegenerativas ou oncológicas na região orofacial e/ou cervical; 3. Estudos envolvendo adultos com fissura labiopalatina, disfagia orofaríngea, síndrome da apneia obstrutiva do sono, disfunção temporomandibular e/ou trauma facial; 4. Estudos secundários, como resumos, revisões sistemáticas, revisões narrativas, revisões integrativas, meta-análises, cartas ao editor, diretrizes e guias de prática clínica; 5. Estudos com amostras repetidas

* A equipe responsável por este estudo entrou em contato, por e-mail, com os autores correspondentes dos estudos excluídos devido ao critério de exclusão 1, com intuito de verificar a formação acadêmica dos mesmos. Apenas o autor Watanabe et al.^10^ respondeu, informando que a equipe era composta por ortodontistas e cirurgiões-dentistas, e não por profissionais formados em fonoaudiologia. Nos demais casos, não houve retorno do e-mail
